# Solving the mystery of vanishing rivers in China

**DOI:** 10.1093/nsr/nwz022

**Published:** 2019-02-11

**Authors:** Yichu Wang, Jinren Ni, Yao Yue, Jiaye Li, Alistair G L Borthwick, Ximing Cai, An Xue, Li Li, Guangqian Wang

**Affiliations:** 1 The Key Laboratory of Water and Sediment Sciences, Ministry of Education, College of Environmental Sciences and Engineering, Peking University, Beijing 100871, China; 2 Beijing Innovation Center for Engineering Science and Advanced Technology, Peking University, Beijing 100871, China; 3 State Key Laboratory of Plateau Ecology and Agriculture, Qinghai University, Xining 810016, China; 4 School of Water Resources and Hydropower Engineering, Wuhan University, Wuhan 430072, China; 5 State Key Laboratory of Hydroscience and Engineering, Tsinghua University, Beijing 100084, China; 6 School of Engineering, The University of Edinburgh, Edinburgh EH9 3JL, UK; 7 Ven Te Chow Hydrosystems Lab, Department of Civil and Environmental Engineering, University of Illinois, Urbana IL 61801, UK

**Keywords:** vanishing rivers, river network, stream segmentation, stream-order rules

## Abstract

A major controversy was sparked worldwide by a recent national water census claiming that the number of Chinese rivers with watersheds ≥100 km^2^ was less than half the previous estimate of 50 000 rivers, which also stimulates debates on the potential causes and consequences. Here, we estimated the number of rivers in terms of stream-segmentation characteristics described by Horton, Strahler and Shreve stream-order rules, as well as their mixed mode for named rivers recorded in the *Encyclopedia of Rivers and Lakes in China.* As a result, the number of ‘vanishing rivers’ has been found to be highly relevant to statistical specifications in addition to the erroneous inclusion of pseudo-rivers primarily generated in arid or frost-thaw areas. The modified Horton stream-order scheme reasonably depicts the configuration of complete natural streams from headwater to destination, while the Strahler largely projects the fragmentation of the named river networks associated with human aggregation to the hierarchical river systems.

## INTRODUCTION

River networks are of primary importance to the continental land mass, water cycle and transportation of materials from land to ocean. In 2013, the First National Census of Water [1] estimated that the number of rivers in China with the catchment area greater than 100 km^2^ was 22 909—a reduction of about 27 000 from the previously estimated figure of over 50 000 rivers in the 1990s [2]*.* This incredible discrepancy also led to international debate [3] as to why rivers are vanishing in China.

Hitherto, tremendous efforts have been made to identify natural river networks from a physical perspective, as hierarchical branching systems. Drainage networks are usually extracted from Digital Elevation Models (DEMs) assuming that water always flows in the steepest downhill direction, and precipitation and geomorphic conditions are uniform [4]. Although these assumptions are generally valid, modification is needed for river networks in arid or semi-arid areas [5]. In practice, the generated drainage networks could be further quantified in terms of different stream-order rules such as the conventional Horton [6], Strahler [7,8] and Shreve [9] schemes, in addition to other options including extrapolation from exemplary basins in the absence of accurate data [2] or statistics from a river encyclopedia [10]. Meanwhile, flow intermittency and hydrologic dis-connectivity of rivers due to climate variability [11,12] and human interventions (e.g. dam construction, urbanization and irrigation) [13–15] over the past decades have considerably increased the complexity of understanding river networks.

Compared with natural river fragmentations, there exists a gap concerning the identification of named river fragmentations. River naming is of fundamental importance to understanding river systems, and the complexity of its identification system derives from various humanity factors [10]. Traditional stream-order schemes, based on the physical structure of rivers, are poorly suited to describing river systems coded with human connotations. To obtain insights into the essential characteristics of river networks, a hybrid stream-order model specifically for estimating the number of named rivers is proposed. This mixed mode, together with other conventional stream-order rules, enables the interpretation of the huge discrepancy between the most well-known estimates of river numbers in China.

## RESULTS AND DISCUSSIONS

### Number of rivers estimated by conventional stream-order rules

From a hydrological perspective, China can be divided into 10 large basins. The drainage networks of those basins are identified from a 30 × 30 m resolution DEMs using a binary search method [16] after pretreatment to remove land surface sinks from the ends of inland rivers (see Supplementary Fig. 1, available as Supplementary Data at *NSR* online). Supplementary Fig. 2, available as Supplementary Data at *NSR* online, shows the resulting hierarchy of drainage networks, of order up to 10. High-order rivers form backbones of the drainage networks, low-order tributaries complete the detailed skeleton.

**Figure 1.**
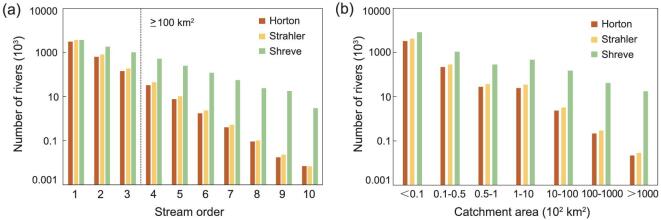
Numbers of rivers in China with (a) different stream orders and (b) different catchment areas estimated by Horton, Strahler and Shreve stream-ordering schemes.

Figure 1a presents the numbers of rivers with different stream orders in China estimated by the Horton, Strahler and Shreve stream-order schemes, respectively. Here, the Shreve orders are reclassified to correspond to the range of the Horton and Strahler orders. For all schemes, the number of rivers diminishes as stream order increases. The results highlight the scheme-dependent statistical characteristics with respect to the number of rivers. For orders 1 to 10, the number reduces from 2 999 117 to 7 using Horton, 3 792 656 to 7 using Strahler and 3 792 656 to 3044 using Shreve. At the lowest order, the river numbers derived from the three schemes are very close; at the high orders, the numbers diverge. Figure 1b presents the numbers of rivers aggregated by catchment areas. For catchment areas from <10 to ≥100 000 km^2^, the number of rivers significantly decreases.

We now focus on rivers in China with the catchment area greater than 100 km^2^, of which about 80% are order 4 to order 10. Supplementary Table 1, available as Supplementary Data at *NSR* online, lists the number of rivers with catchment area ≥100 km^2^ reported in the National Census [1] (*N*_Census_) and estimated by Horton (*N*_Horton_), Strahler (*N*_Strahler_) and Shreve (*N*_Shreve_), respectively. Here, *N*_Horton_ (27 187) provides the closest match to *N*_Census_ (22 909), with a relative difference of about 19%. The Strahler scheme gives an estimate that is 40% higher than that of the National Census. The Shreve scheme provides an estimate that is 29 times larger than *N*_Census_ because of the different stream-ordering schemes used. The similarity between the *N*_Horton_ and *N*_Census_ estimates is unsurprising given that the National Census was implemented using a Horton framework. Discrepancies are evident, resulting from the generation of pseudo-rivers due to the lack of considering factors such as climate, landform and water utilization [5].

### Number of rivers after exclusion of pseudo-rivers

**Figure 2.**
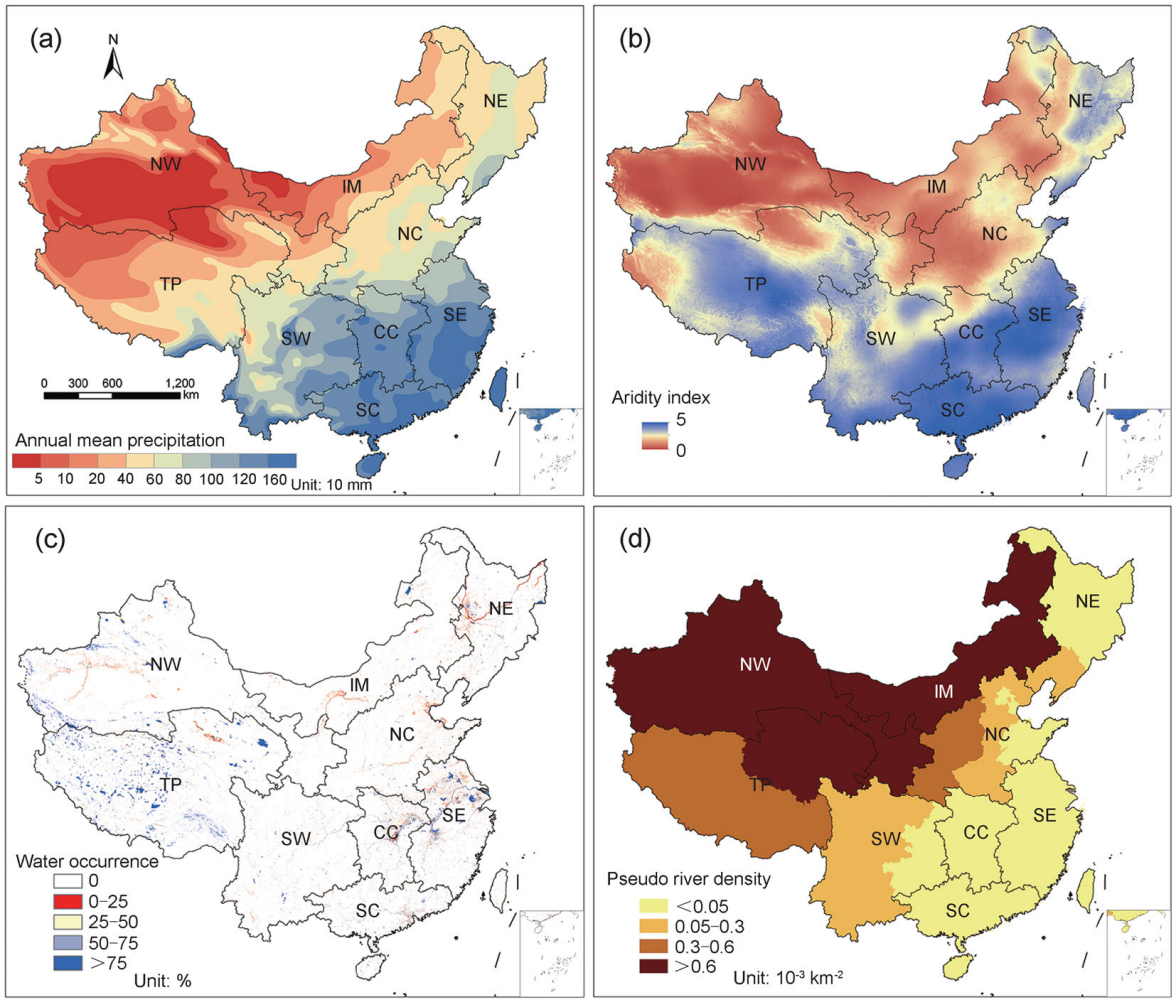
Pseudo-rivers in China. Distribution of (a) annual mean precipitation; (b) aridity index in June, 2011; (c) water occurrence in the period from 1984 to 2015; (d) number of pseudo-rivers with catchment area ≥100 km^2^ per unit area in each province. Here, China is divided into the following nine sub-regions: Northeast China (NE), North China (NC), Central China (CC), Southeast China (SE), South China (SC), Southwest China (SW), Northwest China (NW), Inner Mongolia (IM) and Tibet Plateau (TP).

Exclusion of pseudo-rivers is essential to approach the actual number of rivers approximately reflected by China's First National Census for Water. The pseudo-river could be regarded as a channel without runoff in the wet season. For precipitation-dependent rivers (Fig. 2a), a basin is more capable of producing runoff when it has higher precipitation and lower evaporation [17], coinciding with an aridity index (*AI*) [18] less than certain prescribed threshold values (Supplementary Fig. 3, available as Supplementary Data at *NSR* online; for more details, see Supplementary data, available as Supplementary Data at *NSR* online). Figure 2b maps the distribution of *AI* as it increases from southeast to northwest across China. For basins where rivers are mainly supplied by glacial meltwater and groundwater [19–22], modification is made based on the water occurrence (WO) [23] representing the percentage of available observations in the presence of water during 1984–2015 (Fig. 2c; for more details, see Supplementary data, available as Supplementary Data at *NSR* online). As a result, the total number of rivers (*N*'_Horton_) with catchment area ≥100 km^2^ estimated after the removal of pseudo-rivers (*N*_P_ = ${\rm{4 577}}_{\ {\rm{- 235}}}^{{\rm{\ + 60}}}$) reduces to 22 610, which is only 1.3% less than the Census value (Supplementary Tables 2 and 3, available as Supplementary Data at *NSR* online). Figure 2d maps the density of pseudo-rivers with catchment area ≥100 km^2^ for each province (*D*_P_ (number of pseudo-rivers with catchment area ≥ 100 km^2^ per unit area, 10^−3^ km^−2^)), which equals to *N*_P_ divided by the area of the provinces. *D*_P_ increases from southeast to northwest across China. Pseudo-rivers are mainly located in Northwestern China (NW), Inner Mongolia (IM) and part of the Tibet Plateau (TP) (corresponding to inland, Yellow and international river basins). Inland, Yellow and international river basins contribute 78, 10 and 8% of pseudo-rivers, respectively. In humid regions (i.e. Yangtze, Huai, Pearl river basins and rivers in Zhejiang and Fujian), no pseudo-rivers are identified. Remaining basins (i.e. Amur, Liao and Hai) contribute 4% of pseudo-rivers altogether. About 70% of pseudo-rivers with catchment area ≥100 km^2^ (3150) are generated in arid or frost-thaw areas (i.e. inland river basin and international river basin) due to local violations of the assumptions underpinning runoff simulation (Supplementary Fig. 4, available as Supplementary Data at *NSR* online). The remaining 30% of pseudo-rivers (1427) distributed in North China (NC), Northeast China (NE) and Southwest China (SW) are gullies or dried-out rivers resulting from an arid regional climate with scarce precipitation and strong evaporation, excessive exploitation of water resources or special landform, which accounts for 5.3% of the discrepancy in number of rivers. In the arid and semi-arid areas, the decrease in river discharges in some larger rivers ranged from 7.25 × 10^8^ to 0.48 × 10^8^ m^3^ a^−1^ in recent decades [24–26]. Consequently, some perennial streams have converted to intermittent rivers, even to permanently dry gullies [27]. Long-term overconsumption of water resources also ultimately led to the drying-out of rivers [27,28]. In Yunnan, Guizhou and part of Guangxi province, most precipitation infiltrates directly underground due to the widespread Karst landform, causing many channels to be unable to maintain runoff [29].

After correction of the errors induced by pseudo-rivers, the Strahler scheme gives an estimate (*N*′_Strahler_ = 31 615) that is 38% higher than that of the National Census; the modified Shreve scheme provides an estimate (*N*′_Shreve_ = 557 161) that is 24 times larger than *N*_Census_ (Supplementary Table 1, available as Supplementary Data at *NSR* online) due to the different stream-segmentation characteristics of river networks described by Horton, Strahler and Shreve stream-order rules. However, the influences of incomplete topographic data and landform changes should be fully considered in quantitative studies on river networks (Supplementary Tables 4–8 and Supplementary Figs 5 and 6, available as Supplementary Data at *NSR* online).

### Number of named rivers based on a hybrid stream-order model

Another option for numbering rivers is from records in various geographical chorographies. Historically, river courses have been segmented by random naming that has evolved alongside human culture related to water [10]. However, traditional stream-ordering schemes are not suited to describing river systems fragmented with historical river naming. Here, a systematic comparison is undertaken for 107 river networks (Supplementary Fig. 7, available as Supplementary Data at *NSR* online) listed in the *Encyclopedia of Rivers and Lakes in China* [10] with DEM-generated river networks analysed in terms of different stream-order schemes. We find that arbitrary named river networks can be reasonably replicated using a hybrid of Horton, Strahler and Shreve stream-order schemes. Each basin has a corresponding best-fit proportion in terms of the occurrence possibilities of the three stream-order systems (*P*_f_ = *P*_HO_:*P*_ST_:*P*_SH_) and the most common *P*_f_ is 0.1:0.8:0.1, which occurs with 21 of the 107 representative basins.

In the spirit of the attraction of inhabitants towards rivers [30,31], we find that *P*_f_ for naming river networks is moderately relevant to population density in the year of 1776 during the early Qing dynasty (*D*_1776_) for 107 representative basins (R = 0.4; Supplementary Table 9, available as Supplementary Data at *NSR* online). Thus, six types of basins are classified in terms of *D*_1776_. For each class, the most frequently occurring value of *P*_f_ has been identified (Supplementary Table 10, available as Supplementary Data at *NSR* online) as (*P*_HO_:*P*_ST_:*P*_SH_ = 0.4:0.6:0), (0.3:0.6:0.1), (0.2:0.7:0.1), (0.1:0.8:0.1), (0:1:0) and (0:0.9:0.1), respectively, in basin types I, II, III, IV, V and VI. The population densities are selected from Census information conducted in the year of 1776 during the Qing dynasty, when complete provincial demographic statistics [32] and river-naming systems [33] became commonly established. Since then, the naming system of river networks has remained almost unchanged [10,33] even though China has experienced rapid urbanization [34] and population redistribution [35] in recent decades.

**Figure 3.**
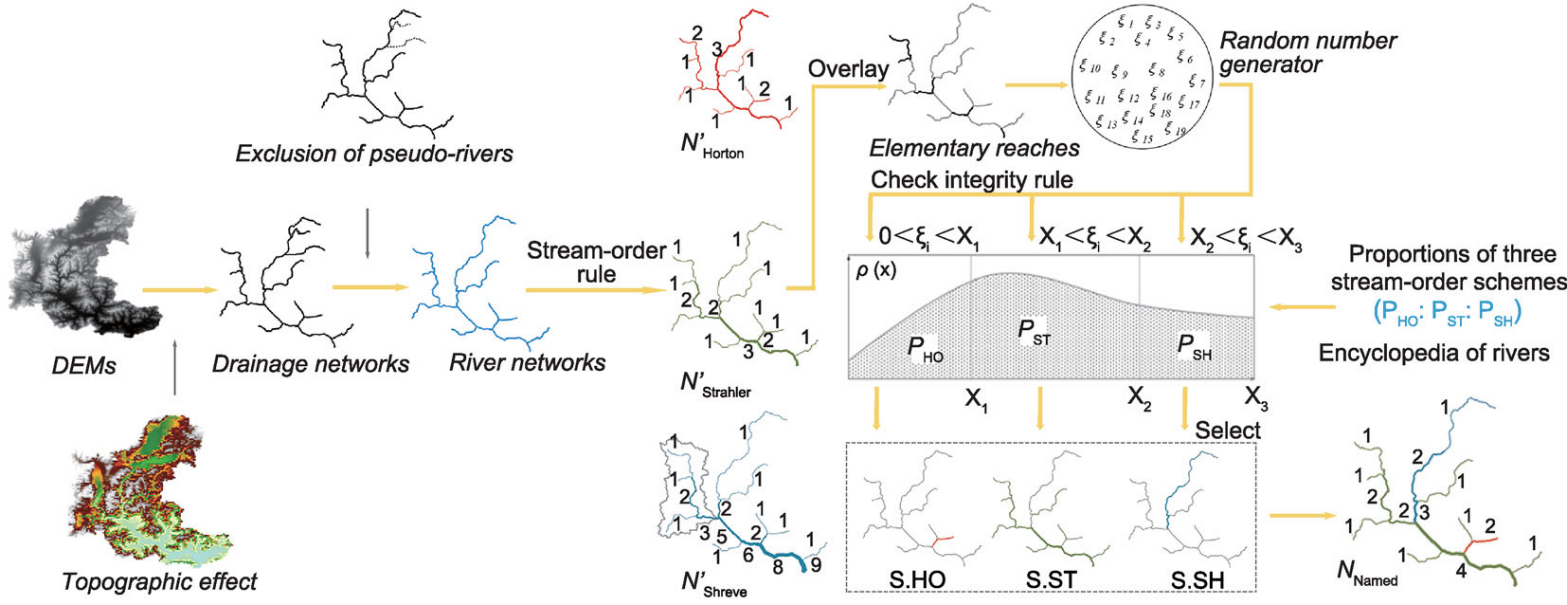
Hybrid stream-order model for estimation of the number of named rivers based on the Monte Carlo method and graph theory.

Having determined *P*_f_ in terms of a human aggregation scale, multiple random tests are then implemented in conjunction with the Monte Carlo method [36], employing a pair-wise scheme for consistency conflict analysis based on a tree structure from graph theory (Fig. 3; for more details, see Supplementary data, available as Supplementary Data at *NSR* online). Taking the above proportions of Horton:Strahler:Shreve as input (Supplementary Fig. 8, available as Supplementary Data at *NSR* online), the total number of named rivers with catchment area ≥100 km^2^ (*N*_Named_) in China in the 1990s is estimated as ${\rm{30\ 201}}_{{\rm{-1187}}}^{{\rm{ + 1299}}}$ (Supplementary Table 11, available as Supplementary Data at *NSR* online), which is ${\rm{7591}}_{{\rm{-1187}}}^{{\rm{ + 1299}}}$ more than that generated solely by the Horton scheme.

**Figure 4.**
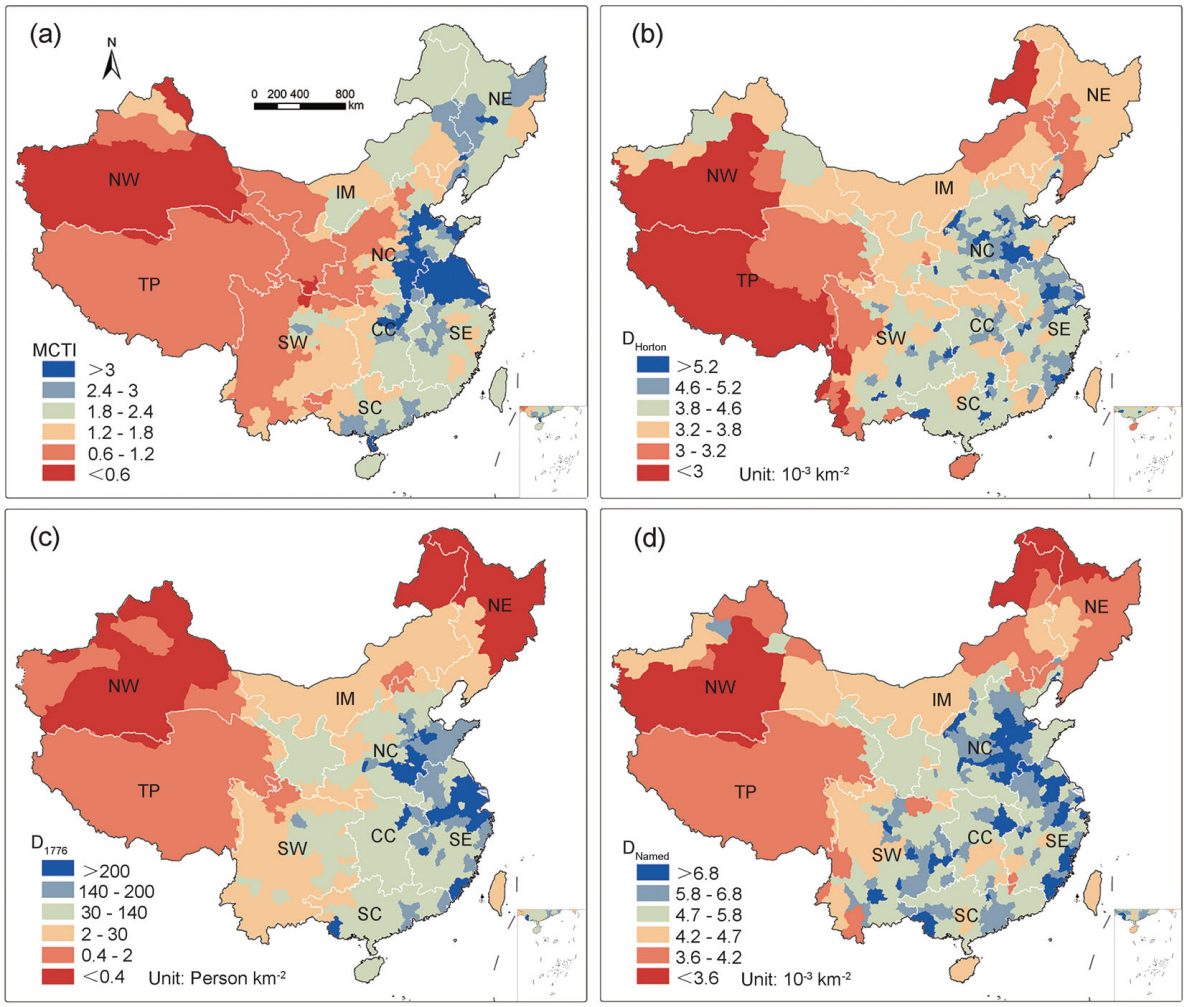
Density of natural rivers and named rivers. Distribution of (a) modified compound topographic index (*MCTI*); (b) natural river density (*D*_Horton_); (c) population density in early Qing dynasty (*D*_1776_); (d) named river density (*D*_Named_).

We further investigated the distribution of both natural river density and named river density in China. Figure 4a maps the distribution of a modified compound topographic index (*MCTI* (a function of surface-flow accumulation, aridity and topographic slope)), which combines surface-flow accumulation, aridity and topographic slope (see Supplementary data, available as Supplementary Data at *NSR* online). *MCTI* shows an apparent decrease from east to west China, with areas of higher *MCTI* corresponding to greater humidity and flatter topography. Figure 4b maps the natural stream frequency distribution (*D*_Horton_ (number of rivers with catchment area ≥ 100 km^2^ estimated by the Horton stream-ordering scheme per unit area, 10^−3^ km^−2^)) throughout China. High values of *D*_Horton_ are distributed in Central China (CC), Southeast China (SE) and NC, with the mean values of 3.26 × 10^−3^, 3.14 × 10^−3^ and 4.11 × 10^−3^ km^−2^. River networks are sparse in NW, SW and TP, with mean values of *D*_Horton_ 2.62 × 10^−3^, 2.93 × 10^−3^ and 2.95 × 10^−3^ km^−2^. It appears that river networks of higher density occur more frequently in humid and flat areas. Figure 4c maps the distribution of population density in the early Qing dynasty (*D*_1776_) throughout China [32]. The most populated regions are located in SE, NC and CC, with mean values of *D*_1776_ 170.26, 98.57 and 80.03 person km^−2^, respectively. Less populated regions are located in TP (1.29 person km^−2^), NE (1.44 person km^−2^) and IM (2.61 person km^−2^). In Fig. 4d, the distribution of the river-naming segment density (*D*_Named_) defining the number of named rivers with catchment area ≥100 km^2^ per unit area is demonstrated. Higher values of *D*_Named_ are found in NC (4.84 × 10^−3^ km^−2^), SE (4.83 × 10^−3^ km^−2^) and South China (SC) and CC (4.52 × 10^−3^ km^−2^), whereas lower values occur in NW (3.28 × 10^−3^ km^−2^), TP (3.60 × 10^−3^ km^−2^) and NE (3.61 × 10^−3^ km^−2^). It appears that the most heavily populated areas coincide with areas that have high densities of named rivers.

### Implications of different statistical specifications

The conventional Horton, Strahler and Shreve stream-order schemes imply different understandings of hierarchical river networks. The Horton scheme [6], describing rivers extending from headwater to outlets, is suitable for understanding the natural configuration of a complete river system. The Strahler scheme [7,8] generates a nested hierarchy of drainage-basin forms, each of which could serve as an open physical system in terms of inputs of precipitation and outputs of runoff, and potentially describes the scale of human aggregation and activities along a large river defined by Horton. The Shreve scheme [9] sums the number of sources in each catchment above a stream gauge or outflow, and is preferred in more accurate considerations in hydrodynamic and source control studies.

**Figure 5.**
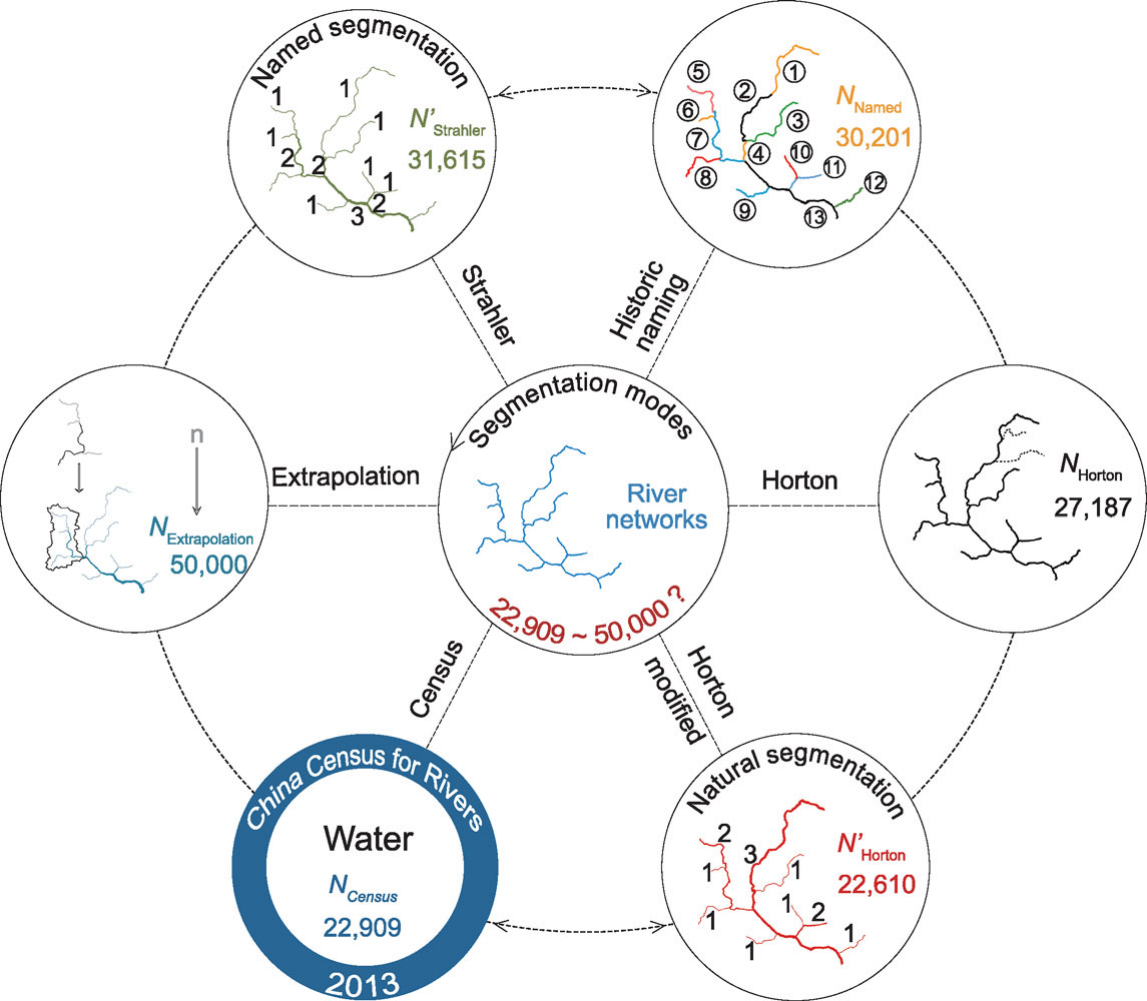
Interpretation of the discrepancy in the numbers of rivers ≥100 km^2^ in terms of representative stream-segmentation modes (outer circles) and their relationship.

In summary, compared with the previous estimate (1990s) of 50 000 rivers ≥100 km^2^ mostly extrapolated from exemplary river networks [2], Fig. 5 indicates a much smaller number of rivers in terms of Horton (22 610), which seems an exact reflection of the natural composition of river networks from headwater to destination described by the latest National Census (22 909) using higher-resolution topographic data (e.g. 1:50 000 DEMs and digital line graphic data) and remote sensing images (e.g. 20 × 20 m CBERS data during 2003∼09 and 2.5 × 2.5 m digital orthophoto map during 2009∼11) [1]. On the other hand, the greater number of rivers derived from the Strahler scheme (31 615) is very close to the number of historically named rivers (30 201), as if a good projection of human aggregation and water culture to the hierarchical river networks. Erroneous inclusion of pseudo-rivers mostly in arid or frost-thaw areas and topographic inputs may also contribute to the huge discrepancy in the number of rivers. Overall, this study is not only of great significance to revealing the mystery of ‘vanishing rivers’ in China in terms of statistical specifications, but also helpful in getting insights into the essentiality of representative river-segmentation modes and their relationship with global river networks.

## METHODS

An efficient method proposed by Bai *et al*. (2015) [16] was adopted for the extraction of drainage networks. For inland rivers, pretreatment is undertaken (Supplementary Fig. 1, available as Supplementary Data at *NSR* online).

Three stream-order schemes including Horton [6], Strahler [7,8] and Shreve [9] stream-ordering schemes are used to describe the hierarchical river networks in this paper.

Pseudo-rivers are identified based on two parameters: AI and WO.

The number of named rivers in China was estimated using multiple random tests in conjunction with the Monte Carlo method [36], employing a pair-wise scheme for consistency conflict analysis based on a tree structure from graph theory.

Details of methods and data sources are given in the Supplementary data, available as Supplementary Data at *NSR* online.

## Supplementary Material

nwz022_Supplemental_File

## Abbreviation List

AI: Aridity Index.
*D*_P_: Number of pseudo-rivers with catchment area ≥ 100 km^2^ per unit area, 10^−3^ km^−2^.
*D*_Horton_: Number of rivers estimated by Horton stream-ordering scheme per unit area, 10^−3^ km^−2^.
*D*_1776_: Population density in Early Qing Dynasty (1776), person km^−2^.
MCTI: Modified compound topographic index, a function of surface flow accumulation, aridity, and topographic slope.
*N*_Census_: Number of rivers with catchment area ≥ 100 km^2^ reported in National Census.
*N*_Horton_: Number of rivers with catchment area ≥ 100 km^2^ estimated by Horton stream-ordering scheme.
*N*'_Horton_: Number of rivers with catchment area ≥ 100 km^2^ estimated by Horton stream-ordering scheme after exclusion of pseudo-rivers.
*N*_Named_: Number of named rivers with catchment area ≥ 100 km^2^.
*N*_P_: Number of pseudo-rivers with catchment area ≥ 100 km^2^.
*N*_Shreve_: Number of rivers with catchment area ≥ 100 km^2^ estimated by Shreve stream-ordering scheme.
*N*'_Shreve_: Number of rivers with catchment area ≥ 100 km^2^ estimated by Shreve stream-ordering scheme after correction of the errors induced by pseudo-rivers.
*N*_Strahler_: Number of rivers with catchment area ≥ 100 km^2^ estimated by Strahler stream-ordering scheme.
*N*'_Strahler_: Number of rivers with catchment area ≥ 100 km^2^ estimated by Strahler stream-ordering scheme after correction of the errors induced by pseudo-rivers.
*P*_ f_: Proportions of three stream-ordering schemes (*P*_HO_: *P*_ST_: *P*_SH_).
*P*_HO_: Probability of occurrence of segments consistent with the Horton scheme.
*P*_SH_: Probability of occurrence of segments consistent with the Shreve scheme.
*P*_ST_: Probability of occurrence of segments consistent with the Strahler scheme.
WO: Water occurrence.
