# Supplementary material for: Solving the mystery of vanishing rivers in China
Source: Natl Sci Rev. 2019 Feb 11;6(6):1239–46. doi: 10.1093/nsr/nwz022 (PMC8291405; doi:10.1093/nsr/nwz022)
Supplement: nwz022_Supplemental_File [file nwz022_supplemental_file.docx]

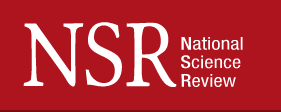


Supplementary Data for

*Solving the mystery of vanishing rivers in China*

Yichu Wang, Jinren Ni, Yao Yue, Jiaye Li, Alistair G. L. Borthwick, Ximing Cai, An Xue, Li Li, Guangqian Wang

Corresponding author: Jinren Ni

Email: [jinrenni@pku.edu.cn](mailto:jinrenni@pku.edu.cn)

**This PDF file includes:**

Supplementary text

Supplementary Figs. 1 to 8

Supplementary Tables 1 to 11

References for SD reference citations

Complete Abbreviation List

**1. Extraction of Drainage networks**

We adopt Bai *et al*.’s (2015) [1] compound method to extract drainage networks. This computationally efficient method comprises five steps: I. pretreatment to fill local depressions; II. determination of flow directions; III. accumulation of upslope areas; IV. extraction of drainage channel; and V. vectorization of geographic features (Supplementary Fig. 1).

For interior rivers, pretreatment is undertaken. Lakes and deserts act as sinks for inland rivers. Lake sinks are identified by overlaying information from the global lake and wetland database (GLWD) [2] on the DEMs. The GLWD data relate to lakes of area larger than 0.1 km^2^, and so only sinks of area ≥ 0.1 km^2^ are identified. The remaining rivers are considered to end in the desert. For these basins we set a critical elevation value based on the Hydrosheds dataset [3], below which a region is a sink. After identifying the sinks, we use Arc info to erase the sink pixels. Thus, the sinks of inland rivers form the boundaries of the DEMs in a similar way to the sinks of exorheic rivers.

The extracted drainage networks were compared with the Hydrosheds Data [3]. And it appears that location of major rivers (stream order ≥ 6) is consistent with that derived from the Hydrosheds Data.

The hierarchical drainage networks which are extracted from 30 m resolution Aster GDEMs based on the above method for ten basins of China are illustrated in Supplementary Fig. 2.

**2. River system hierarchy**

Three stream-order schemes including Horton, Strahler, and Shreve stream-ordering schemes are used to describe the hierarchical river networks in this paper.

*2.1 Horton stream-ordering scheme*

In applying the Horton scheme [4], we first specify the maximum stream order, $\text{n}_{\text{max}}$. The river corresponding to stream order *n* = *n*_max_ is the longest drainage path in the basin, and hence forms the backbone of the river network. The remainder of the network is then ordered into river bifurcations, at progressively lower orders until *n* = 1 is reached and the finest-scale rivers with shortest drainage paths leading from higher order river segments have been identified. Rivers of *n*-th stream order are identified by searching for the longest drainage paths that connect to the *n*+1-th order rivers. This procedure is repeated for *n* = *n*-1, *n*-2, ... 1. This method gives exactly the same results for river segmentation as the original Horton scheme even though its implementation is slightly different.

It should be noted that based on each stream ordering scheme, rivers can also be classified by catchment area. For example, in China’s First National Census of Water [5], the number of rivers with catchment area ≥ 10,000 km^2^, 1,000–10,000 km^2^, 100–1,000 km^2^, and 50–100 km^2^ are estimated separately, based on the Horton scheme. And Supplementary Table 2 displays the number of rivers (and pseudo rivers) aggregated by catchment areas based on the Horton scheme identified from 30 m × 30 m DEMs. The number of rivers decreases as catchment area increases, for catchment area 10–50 km^2^, to ≥ 100,000 km^2^ the number reduces from 215,385 to 23.

*2.2 Strahler stream-ordering scheme*

In the Strahler scheme [6, 7], first-order rivers have headwaters without bifurcation. And when there is a confluence, the stream-order of downstream rivers obeys the following rules:

$\text{n}_{\text{d}}\text{ = }\left\{ \begin{aligned} \text{n}_{\text{1}}\text{+1, for }\text{n}_{\text{1 }}\text{= }\text{n}_{\text{2}} \\ \text{max(}\text{n}_{\text{1}}\text{, }\text{n}_{\text{2}}\text{),}{\text{ for }\text{n}}_{\text{1}}\text{ }\text{≠}\text{ n}_{\text{2}} \end{aligned} \right.$ (1)

where *n*_d_ is the stream order of the downstream river, and *n*_1_ and *n*_2_ are the respective stream orders of the upstream rivers at the bifurcation.

*2.3 Shreve stream-ordering scheme*

In the Shreve scheme [8], the first-order rivers are headwaters without bifurcation, and form the branches at the finest level of the network. At a confluence, the stream order of the downstream river is the sum of the orders of the two upstream rivers.

**3. Identification of pseudo-rivers**

*3.1 Pseudo-river identification hypothesis*

We cannot ensure that channels identified via DEMs are in fact rivers because, on the one hand, the drainage networks extraction method is not totally suitable for the rivers in arid areas [9], and on the other hand, the topographic data do not contain information on local climate conditions, soil characteristics, vegetation cover, etc [10, 11]. To make direct comparison with number of rivers reported in China’s First National Census for Water [5], modification is needed by excluding pseudo-rivers from the extracted drainage networks. The most obvious difference between a real river and a pseudo-river is determined by the runoff condition in the channel. From the climate perspective, a basin is more capable of producing runoff when it has higher precipitation and lower evaporation [12]. Thus, the following pseudo-river identification hypothesis is proposed, based on common sense. A pseudo-river is defined as a channel that has zero runoff in the wet season and is located in a river basin whose Aridity index (*AI*) [13] is less than a prescribed threshold value.

Based on the standard dry-wet climate classification [14], the threshold value is taken to be 0.2, at the boundary between arid and semi-arid zones. When *AI* = 0.2, the ratio of actual evaporation to precipitation is equal to unity according to the Budyko Curve (Supplementary Fig. 3) [15-18]. This means all precipitation is converted into evaporation, and no runoff is generated, and is in accordance with findings from previous studies that a dry climate in semi-arid and arid zones promotes the development of pseudo-rivers [19, 20]. The proposed hypothesis is mainly suitable for rivers that are supplied by rainfall, and so for inland basins, international river basins, and the Yellow River basin where rivers are mainly supplied by glacial meltwater and groundwater [21-24], modification is carried out, based on the Water Occurrence (*WO*) [25] parameter which represents water dynamics from 1984 to 2015 expressed as a percentage of available observations when water is present. Specifically, we identify a channel as a pseudo-river when any element in the channel’s series of water occurrence values between 1984 and 2015 is zero. Moreover, noting that large rivers with catchment area ≥ 1,000 km^2^ always have stable runoff [26], we focus solely on rivers with catchment area < 1,000 km^2^ during the identification process. Based on *AI* and *WO*, 4,577 pseudo-rivers with catchment areas ≥ 100 km^2^ (*N*_P_) within the main river basins are identified (Supplementary Table 3).

To eliminate the influence of pseudo-rivers for *N*_Strahler_ and *N*_Shreve_, we use the ratio of pseudo-rivers ($\text{R}_{\text{P}}$) to revise the results:

$\text{R}_{\text{P}}\text{ =}{\text{N}_{\text{P}}}/{\text{N}_{\text{Horton}}}$ (2)

${\text{N}\text{'}}_{\text{Stra}\text{h}\text{ler}}\text{ = }\text{N}_{\text{Strahler}}\text{ ×}\text{ }\text{(1}-{\text{ }\text{R}}_{\text{P}}\text{)}$ (3)

${\text{N}\text{'}}_{\text{S}\text{hreve}}\text{ = }\text{N}_{\text{S}\text{hreve}}\text{ ×}\text{ }\text{(1}-{\text{ }\text{R}}_{\text{P}}\text{)}$ (4)

The revised number of rivers ≥ 100 km^2^ estimated by Horton, Strahler, and Shreve stream-order scheme (${\text{N}\text{'}}_{\text{Strahler}}$, ${\text{N}\text{'}}_{\text{Strahler}}$, ${\text{N}\text{'}}_{\text{Shreve}}$) for ten basins of China is displayed in Supplementary Table 1.

*3.2 Constituent analysis of pseudo-rivers*

Pseudo-rivers generally occur where the runoff simulation assumption is invalid, gullies, and dried-out rivers. Now we examine the contributions of these two types of pseudo-rivers in turn.

The runoff simulation assumption is often invalid in non-water erosion areas (Supplementary Fig. 4) where both precipitation and the underlying geomorphic condition are non-uniform [19]. We find the number of pseudo-rivers ≥ 100 km^2^ distributed in non-water erosion areas (<http://cese.pku.edu.cn/chinaerosion/>) of China is 3,150, which is 69% of the total number of pseudo-rivers.

By subtracting the number of pseudo-rivers which generally occur where the runoff simulation assumption is invalid, and dried-out rivers from the total number of pseudo-rivers, we obtain the number of gullies and dried-out rivers ≥ 100 km^2^ of China to be 1427, which is 31% of the total number of pseudo-rivers. Gullies and dried-out rivers are mainly located in Loess Plateau, Karst landform areas, in addition to Liao, Amur, and Hai River basin.

*3.3 Error analysis and sensitivity analysis*

By comparing the values of $\text{N}_{\text{Horton}}\text{'}$ and $\text{N}_{\text{Census}}$ for ten basins of China (Supplementary Table 1), we find the mean error range for *N*_P_ is ± 5.5%. This error partly arises from the -1%–5% error in the water occurrence dataset, allowing for 1% false water detections and 5% missing data [25]. So the total number of pseudo-rivers with catchment area ≥ 100 km^2^ for the Yellow, inland, and international river basins ranges from 4,162 to 4,425. Meanwhile, there is an 8% error in the aridity index, so the total number of pseudo-rivers with catchment area ≥ 100 km^2^ for the remaining seven basins ranges from 180 to 212. Summing these two parts together, the number of pseudo-rivers ≥ 100 km^2^ is estimated to be ${\text{4,}\text{577}}_{\text{-}\text{235}}^{\text{ }\text{+}\text{60}}$.

A sensitivity analysis is conducted to test whether 0.2 is reasonable for the threshold value of aridity index. In addition to the Census information and the original study, five further scenarios are considered, with threshold values of 0.1, 0.15, 0.2, 0.25, 0.3, respectively. It should be noted that Scenario 3 corresponds to the case in Section 3.1. The exclusion rates of pseudo rivers with catchment area ≥ 100 km^2^ for Yangtze, Amur, Liao, Hai, Huai, and Pearl river basins, and rivers in Zhejiang and Fujian for Scenarios 1, 2, 3, 4, and 5 are 0.2%, 0.4%, 1.5%, 3.1%, and 5.9%, respectively. Thus, the pseudo-river exclusion rates for these basins are not sensitive to the threshold value of *AI.*

*3.4 Extrapolation of results*

Supplementary Table 3 also displays the number of pseudo-rivers aggregated according to catchment area. The number of pseudo-rivers decreases with basin area. Small rivers are in a more dynamic hydrologic condition because they are more sensitive to precipitation [26].

**4. Incomplete topographic data**

*4.1 Discrepancy in numbers of rivers obtained from different topographic data*

Moreover, the influences of incomplete topographic data and changes to land surface topography should also be fully considered in quantitative studies on river networks. Another two DEM datasets were used to investigate the effect of DEM resolution on numbers of rivers.

Supplementary Figure 5 displays the numbers of rivers of China aggregated by catchment areas extracted from 900 m × 900 m (*N*_900_), 90 m × 90 m (*N*_90_), and 30 m × 30 m DEMs (*N*_30_). We find for large rivers (catchment area ≥ 1000 km^2^), *N*_900_, *N*_90_, and *N*_30_ are similar. The relative difference between *N*_90_ and *N*_30_ maintain at the values of about 5% until the catchment areas reduces to 50 km^2^. For rivers with catchment area < 50 km^2^, the relative difference between *N*_90_ and *N*_30_ reaches 10% (Supplementary Table 4). However, the low-resolution topographic data in the 1990s can only identify large rivers, and fails fully to identify small rivers (catchment area < 1000 km^2^). For small rives with catchment area < 1000 km^2^, the relative difference between *N*_900_ and *N*_30_ is as much as 30% (Supplementary Table 5). Overall, it appears that the 90 m × 90 m DEMs in the 2000s can identify rivers with catchment area ≥ 50 km^2^, while the low-resolution topographic data of the 1990s (900 m × 900 m DEMs) are only suitable for identification of large rivers with catchment area ≥ 1000 km^2^.

One approach to calculating the number of rivers ≥ 100 km^2^ in the 1990s would be to extrapolate from the number of large rivers. Here, we calculate the number of rivers of area < 1,000 km^2^ in the 1990s by extrapolation from the number of rivers ≥ 1,000 km^2^. For a Horton-Strahler self-similar drainage network, we calculate the number of rivers at each order via *R*_B_ [27]. Here we assume that when a Horton scheme transforms into a catchment area scheme, the drainage network is also self-similar, and there is a pseudo-bifurcation ratio (called $\text{R}_{\text{B}}\text{'}$) between rivers with different catchment area grades. Here it is not possible to obtain the pseudo-bifurcation ratio using the least-squares method [4], due to incomplete identification of small rivers. Here we adopt another method to approximately calculate the pseudo-bifurcation ratio ($\text{R}_{\text{B}}\text{'}$) [28] for the ten basins of China, defined as:

*R*_B_' = *N*_900(1000-10000)_ / *N*_900(10000-100000)_ (5)

*R*_B_" = *N*_30(50-100)_ / *N*_30(100-1000)_  (6)

Thus,

*N*_900(100-1000)_ = *N*_900(1000-10000)_ × *R*_B_' (7)

*N*_900(10-100)_ = *N*_900(1000-10000)_ × (*R*_B_')^2^ (8)

*N*_900(50-100)_ = *N*_900(1000-10000)_ ×(*R*_B_') × (*R*_B_") (9)

*N*_900(10-50)_ = *N*_900(1000-10000)_ × (*R*_B_')^2^– *N*_900(1000-10000)_ ×(*R*_B_') × (*R*_B_") (10)

*N*_T_= *N*_900(100-1000)_ + *N*_900(1000-10000)_ + *N*_900(10000-100000)_ + *N*_900(≥100000)_ (11)

*N*_900(≥100000)_, *N*_900(10000-100000)_, *N*_900(1000-10000)_, *N*_900(100-1000)_, *N*_900(50-100)_, *N*_900(10-50)_, *N*_900(10-100)_ represent the numbers of rivers with catchment areas ≥ 100,000 km^2^, 10,000–100,000 km^2^, 1,000–10,000 km^2^, 100–1000 km^2^, 50–100 km^2^, 10–50 km^2^, and 10–100 km^2^ extracted from 900 m × 900 m DEMs, respectively. *N*_30(50-100)_, *N*_30(100-1000)_ are numbers of rivers with catchment areas 50–100 km^2^, 100–1000 km^2^. *N*_T_ is the number of rivers with catchment area ≥ 100 km^2^ extrapolated from 900 m × 900 m DEMs.

Based on equation (5), (6), the value for *R*_B_', and *R*_B_" of China is 10.9, and 1.1, respectively. By inserting $\text{N}_{\text{900(≥100,000)}}$, $\text{N}_{\text{900(10,000-100,000)}}$, $\text{N}_{\text{900(1,000-10,000) }}$for ten basins of China, *R*_B_', and $\text{R}_{\text{B}}\text{''}$ into equations (7) – (11), we obtain values for estimated *N*_900_ with different catchment areas, and *N*_T_ (Supplementary Table 6).

The resulting number of rivers ≥ 100 km^2^ extrapolated from low-resolution topographic data ($\text{N}_{\text{T}}$) is ${\text{28,25}\text{0}}_{\text{-}\text{13418}}^{\text{+}\text{7297}}$, about 1,063 more than the result based on 30 m × 30 m DEM ($\text{N}_{\text{Horton}}$). Previous research has suggested that use of a low-resolution DEM causes overestimates of river catchment areas [29], providing further evidence for the overestimate of the number of small rivers after extrapolation.

*4.2 Error Analysis*

Noting that $\text{R}_{\text{B}}\text{'}$ has a range of 5.2 ~ 14.0, then *N*_T_ also has a corresponding range. Inserting both *R*_B_' = 4.2, and *R*_B_' =14.0 into equation (7)– (10), we obtain the minimum value, and maximum value of $\text{N}_{\text{T}}$ of China to be 14832, and 35547, respectively (Supplementary Table 6).

**5. Changes to land surface topography**

*5.1 Discrepancy in the number of rivers due to change in topography*

During the recent two decades, channel geometries and river network topologies have altered due to intensive urbanization and rapid land-use changes in China, which may also cause the extraction of river networks to vary [30, 31**]**. Here we used GTOPO30DEMs (900 m × 900 m, launched in 1996) and AsterGDEMs (30 m × 30 m, launched in 2011) to represent China’s land surface topography in the 1990s and 2010s, respectively. During this period, China experienced intensive urbanization and land-use changes [32]. Drainage networks extracted from each DEMs are compared to explore the effect of network alteration caused by topographic changes on the number of rivers. To minimize the effect of DEMs resolution, we re-sampled 30 m AsterGDEMs at 900 m. The re-sampled DEMs is called 900 m DEMs' herein.

Let ${\text{N}\text{ }}_{\text{900}}$ and ${\text{N}\text{ }}_{\text{900}}$' be the numbers of rivers with catchment area ≥ 100 km^2^ identified from the 900 m DEM and 900 m DEMs'. Supplementary Table 7 lists values of ${\text{N}\text{ }}_{\text{900}}$' with different catchment areas. Comparing ${\text{N}\text{ }}_{\text{900}}$ with *N*_900_', we find relative difference between *N*_900_' and *N*_900_ increase from -0.2 % to -14% when the catchment area of rivers decreases from 1,000-100,000 km^2^ to 10-50 km^2^. It means the topographic change mainly cause variation of number of small rivers instead of large rivers.

Here we assume that both the rivers identified by 900 m DEMs' and those not identified by 900 m DEMs' contain the same percentage of migrated rivers ($\text{P}_{\text{M}}$). Thus, we approximately estimated the difference in numbers of rivers with catchment area ≥ 100 km^2^ due to topographic change (*N*_M_):

$\text{N}_{\text{M}}\text{ }\text{= }{\text{N}\text{ }}_{\text{900}}'-\text{ }{\text{N}\text{ }}_{\text{900}}\text{ + }\left( {\text{N}\text{ }}_{\text{30}}-{\text{N}\text{ }}_{\text{900}}' \right)\text{ }\text{×}\text{ }\text{P}_{\text{M}}$ (12)

*P*_M_ = (*N*_900_' – *N*_900_) / *N*_900_  (13)

*P*_M_ is the percentage of migrated rivers ($\text{P}_{\text{M}}$).

Based on equation (12), (13), we also obtain the difference in numbers of rivers with catchment areas ≥ 100 km^2^ for ten river basins of China (Supplementary Table 8).

It appears that changes in land topography from 1996 to 2011 caused the number of rivers with catchment area ≥ 100 km^2^ ($\text{N}_{\text{M}}$) to decrease by 1953, which accounts for 7.2% of the number of “vanishing rivers”. The difference in numbers of rivers due to topographic change reduces across eastern and central China except for a slight increase in the Hai River and Huai River Basin (Supplementary Table 1) owing to the construction of artificial channels [33]. The topographic changes in elevation during 1996–2010 are concentrated in the Hai, Huai, Pearl River basin, rivers in Zhejiang and Fujian, and estuary regions of the Yangtze River and Yellow River basins (Supplementary Fig. 6) which comprise relatively flat (Supplementary Table 8 lists mean slopes), urbanized areas.

*5.2 Error Analysis*

Since $\text{P}_{\text{M}}$ has a range of -4.3% – 13.7% (Supplementary Table 8), then *N*_M_ also has a corresponding range. Inserting both $\text{P}_{\text{M}}$= -4.3% and $\text{P}_{\text{M}}$= 13.7% into equation (12), gives $\text{N}_{\text{M}}$=${\text{ 1}\text{,}\text{953}}_{\text{-}\text{958}}^{\text{+}\text{623}}$.

**6. Random river naming**

*6.1 Hybrid Stream-Order Scheme based on Monte Carlo method*

Drainage networks are split into segments following prescribed rules when using a river-ordering scheme. Based on numerous comparisons of the named river systems (*Encyclopedia of Rivers and Lakes in China* [34]) and river networks generated using the three different stream-order rules for various-scale basins in China, we found the historical river naming practice was best replicated by a mix of Horton, Strahler, and Shreve ordering schemes. This implies the number of rivers in a named river system can be simulated using different stream-order schemes in different regions. However, no information can be gleaned from historical records about the spatial distribution of river reaches determined by each of the three schemes. Consequently, the number of historically named rivers in China can only be assessed by combining information about the probabilities of occurrence of usage of the three stream-order schemes, which is approximated by recorded frequencies of occurrence in the sample watersheds in the *Encyclopedia of Rivers and Lakes in China* [34]. By intersecting different layers of river networks according to the stream-order schemes of Horton, Strahler, and Shreve, a set of elemental river reaches is generated. Then, assessments by the three stream-order schemes are distributed according to their occurrence probabilities to each of the river reaches, and the total number of river reaches estimated using the hybrid stream-order model.

To avoid the subjective error that may be incurred by manually assigning a stream-order scheme to a given element reach, the Monte Carlo method [35] is implemented to simulate the random choice of stream-order scheme. This method also enables scenario analysis by assigning different combinations of occurrence probabilities to the three stream-order schemes. The core idea behind the Monte Carlo method is to simulate a random process according to an appropriate statistical model, and then to approximate the true solution by a suitable estimate. Fig. 3 displays the framework of this naming system. The procedure underpinning the naming system is as follows:

(I) Estimate probabilities of occurrence of Shreve, Strahler, and Horton river reaches (i.e. *P*_HO_, *P*_ST_, *P*_SH_) by analyzing occurrence frequencies of the three stream-order schemes in the sample watersheds considered in the *Encyclopedia of Rivers and Lakes in China* [34].

(II) Select an appropriate probability density function, *f*(*x*).

(III) Set up correspondence between the three stream-order schemes and different intervals of *f*(*x*), which meets the following requirements:

$\left\{ \begin{aligned} \text{P}_{\text{HO}}\text{=}\int_{\text{0}}^{\text{X}_{\text{1}}} \text{f}\left( \text{x} \right)\text{d}\text{x} \\ \text{P}_{\text{S}\text{T}}\text{=}\int_{\text{X}_{\text{1}}}^{\text{X}_{\text{2}}} \text{f}\left( \text{x} \right)\text{d}\text{x} \\ \text{P}_{\text{S}\text{H}}\text{=}\int_{\text{X}_{\text{2}}}^{\text{X}_{\text{3}}} \text{f}\left( \text{x} \right)\text{d}\text{x} \end{aligned} \right.$ (14)

Suppose that *I(x)* is the integral function of *f(*x*)*:

$\text{I}\text{(}\text{x}\text{)}\text{ }\text{=}\int_{\text{0}}^{\text{x}} \text{f}\text{(}\text{x}\text{)dx}$ (15)

Then, X_1_, X_2_, and X_3_, which define the intervals of *f*(*x*), can be determined as:

$\left\{ \begin{aligned} \text{X}_{\text{1}}\text{=}\text{I}\text{(}\text{X}_{\text{1}}\text{)} \\ \text{X}_{\text{2}}\text{=}\text{I}\left( \text{X}_{\text{2}} \right)\text{-}\text{X}_{\text{1}} \\ \text{X}_{\text{3}}\text{=}\text{I}\left( \text{X}_{\text{2}} \right)\text{-}\text{X}_{\text{2}} \end{aligned} \right.$ (16)

(IV) Set up a pseudo-random number generator to simulate a uniformly distributed random variable, *ξ*, within the range from 0 to X_3_. The naming scheme is as follows:

When 0 < *ξ* ≤ X_1_, the element reach of interest is selected as a Horton river;

When X_1_ < *ξ* ≤ X_2_, the reach is classified as a Strahler river; and

When X_2_ < *ξ* ≤ X_3_, the reach is chosen as a Shreve river.

Integrate *f*(x) as:

$\text{P}_{\text{i}}\text{=}\int_{\text{Xj-1}}^{\text{Xj}} \text{f}\left( \text{x} \right)\text{d}\text{x}\text{, (}\text{i}\text{ }\text{=}\text{ }\text{Horton}\text{, }\text{Stra}\text{h}\text{ler}\text{, }\text{Shreve}\text{;}\text{ j}\text{ }\text{=}\text{ }\text{1, 2, 3; }\text{X}_{\text{0}}\text{ }\text{=}\text{ }\text{0)}$ (17)

Define another random variable, *η* = *f(ξ)*. Then,

$\text{P}_{\text{i}\text{n}_{\text{i}}}\text{=}\frac{\sum_{\text{1}}^{\text{n}_{\text{i}}} \text{η}_{\text{j}}}{\text{n}_{\text{i}}}\text{=}\frac{\sum_{\text{1}}^{\text{n}_{\text{i}}} {\text{f}\text{(}\text{ξ}}_{\text{j}}\text{)}}{\text{n}_{\text{i}}}$ (18)

Hence,

$\text{P}_{\text{i}\text{n}_{\text{i}}}\text{=}\text{E}\left( \text{η} \right)\text{=}\lim_{\text{n}_{\text{i}}\text{→∞}} \text{P}_{\text{i}\text{n}_{\text{i}}}$ (19)

Which implies that $\text{P}_{\text{i}\text{n}_{\text{i}}}$ is the unbiased estimator of *P*_i_ when *n*_i_ is sufficiently large.

(V) Assign IDs to each of the integrated river units in terms of the three stream-order schemes, which implies each elemental river reach has three IDs (namely, *ID*_Horton_, *ID*_Strahler_, and *ID*_Shreve_) derived from overlay of the three river networks.

(VI) Generate integrity information and marked information for each of the elemental river reaches.

(VII) Randomly assign a stream-order scheme according to IV, and check if the assignment is valid according to the flags in the marked system. For valid assignment, simply change the marked information of other elements in the same basic unit of rivers to that of the assigned scheme.

(VIII) By defining the function, *ID*_(ni)_, to return the total number of distinct *ID*s (i = Horton, Strahler, and Shreve, as in Step (IV)), the Monte Carlo estimate of the total number of river reaches, *N*_MC_, is as follows:

$\text{N}_{\text{MC}}\text{=}\sum_{\text{Horton}\text{, St}\text{rahler}\text{, }\text{Shreve}} \text{ID}\text{(}\text{n}_{\text{i}}\text{)}$ (20)

(IX) Repeat Steps (IV) and (VIII) M times, and we can obtain the series of *N*_MC_s: *N*_MC-1_, *N*_MC-2_, ……, *N*_MC-M_. The mean *N*_MC_ is:

$\bar{\text{N}_{\text{MC}}}\text{=}\frac{\sum_{\text{1}}^{\text{M}} \text{N}_{\text{MC-j}}}{\text{M}}$ (21)

According to the law of large numbers, the actual number of river reaches, *n*_M_, is:

$\text{n}_{\text{M}}\text{=}\lim_{\text{M→∞}} \overline{\text{N}_{\text{MC}}}$ (22)

Which means that the mean Monte Carlo estimator approaches *n*_M_ for sufficiently large numbers of simulations.

*6.2 Proportions of the three ordering schemes in the named river systems*

When simulating the named river system using the Monte Carlo method, the first step is to obtain the proportions of the three stream-ordering schemes ($\text{P}_{\text{f}}$). To quantify suitable values for $\text{P}_{\text{f}}$ in the named river system, we collect data on 107 representative basins whose catchment areas range from 2,690 km^2^ to 137,633 km^2^ (Supplementary Fig. 7) from *Encyclopedia of Rivers and Lakes in China* [34]. For each basin, best-fit segment proportions are determined for the three stream-ordering schemes. The procedure for determining $\text{P}_{\text{f}}$ for each basin is as follows:

1. Vectorize the basin’s drainage network map, obtained from the *Encyclopedia of Rivers and Lakes in China*.
2. Create a series of elemental reaches for the river network by splitting the rivers at their vertices; the total number of elemental reaches in the series is *n*_Total_.
3. Name each elemental reach according to information extracted from the *Encyclopedia of Rivers and Lakes in China*.
4. Compare the resulting named river system with the river network defined by the Strahler stream-ordering scheme. Assign a value of 2 to named elementary reaches that are consistent with the Strahler scheme.
5. Consider those named reaches that are not consistent with Strahler. Then compare these reaches with results from the Shreve and Horton stream-ordering schemes, and assign a value of 1 to those consistent with Horton, and 3 to those consistent with Shreve. It should be noted that if a reach is assigned the value 1, its downstream reaches with the same Horton order should also be assigned the value 1 (according to the Horton ordering rule, and overwriting any previously assigned values).
6. Total numbers of reaches assigned values 1, 2, and 3, are denoted *n*_HO,_ *n*_ST_, and *n*_SH_.
7. Segment proportions for the Horton (*P*_HO_*)*, Strahler (*P*_ST_), and Shreve (*P*_SH_) schemes are evaluated from *n*_HO_ */ n*_Total_, *n*_ST_ */ n*_Total_, and *n*_SH_ */ n*_Total_.

The reason why we first compare the named river system with rivers defined by Strahler ordering scheme in Step (4) is that the Strahler scheme gives results closest to those from traditional named river segmentation.

The use of 107 sub-basins to represent the drainage behavior of their parent basins is acceptable given that: (i) they cover 26% of China’s total land area; (ii) they cover most geomorphic types in China including loess, fluvial landform, karst, and desert; (iii) their surface morphology ranges from flat plain to very steep mountains; and (iv) their aridity indices range from semi-arid to hyper-humid.

*6.3 Method validation*

After determining $\text{P}_{\text{f}}$ for 107 representative basins, we compute the number of named rivers using random simulation based on the Monte Carlo method for each basin (*n*_Mixed_) in order to validate the model. We denote the relative difference between *n*_Mixed_ and *n*_Named_ for the 107 representative basins as *Dif*, There are 31, 42, and 34 basins with values of *Dif* equal to zero, > zero, and < zero, respectively, which implies the error is random. Meanwhile, 93 basins have 87% of their *Dif* values lying between -10% and 10%, which confirms the model has satisfactory accuracy.

*6.4 Correlation between river naming and population density*

The naming of rivers is essentially a human activity, and so we assume the river naming process is also associated with population density. To confirm the validity of this assumption, a correlation analysis would normally be required. However, the proportions of the three schemes ($\text{P}_{\text{f}\text{ }}$) which represent the naming orientation of rivers cannot be directly fitted to a correlation analysis. In such cases, we replace $\text{P}_{\text{f}}$ with ${\text{N}\text{P}}_{\text{f}}$.

We consider 66 scenarios with different proportions of the Horton, Strahler, and Shreve river-ordering schemes, and compute the total number of rivers *N*_Mixed_ of China for each scenario, using random simulation based on the Monte Carlo method (Fig. 3). Supplementary Table 9 is a ranked list in order of the least value to the highest value of *N*_Mixed_, in which each scenario is signed a number. Here, $\text{N}_{\text{Mixed}}$ is quite insensitive to the relative proportions of Horton and Strahler, when the Shreve proportion is small ($\text{NP}_{\text{f}}$ 1 to 20). $\text{N}_{\text{Mixed}}$ is highly sensitive to the proportion of the Shreve stream-ordering scheme, and increases substantially for Scenarios 56–66. It is interesting to note that when the drainage network is composed of 10 % rivers defined by Horton, 20% rivers defined by Strahler, and 70 % rivers defined by Shreve, the total value of $\text{N}_{\text{Mix}\text{ed}}$ for China reaches ~ 50,000 (the textbook value at the center of the recent controversy). Altering the mix further, it is possible to obtain 669,946 rivers when Shreve is 1, and Horton and Strahler are both zero.

The value of ${\text{N}\text{P}}_{\text{f}}$ increases with *N*_Mixed_. Since *N*_Mixed_ is the number of named rivers, a high value of $\text{NP}_{\text{f}}$ corresponds to a high frequency of naming of rivers. A correlation has been determined between the Scenario ${\text{N}\text{P}}_{\text{f}}$ and the population density in Qing Dynasty (1776) [36] for 107 representative basins. The analysis shows that Scenario ${\text{N}\text{P}}_{\text{f}}$ and the population density in Qing Dynasty (1776) are positively correlated (*R* = 0.4, *P* = 0.01), confirming that the assumption is reasonable.

*6.5 The number of named rivers in China*

We separately compute the local $\text{N}_{\text{Named}}$ of each of the four population modes using the corresponding proportions of the three ordering schemes (Supplementary Table 10), then sum the values together. The local $\text{N}_{\text{Named}}$ values for basins of type I, II, III, IV, V, and VI are 6453, 8697, 9155, 8594, 1811, and 1605, respectively (Supplementary Fig. 8). In this way, we obtain $\text{N}_{\text{Named}}$ for China to be $\text{36,315}_{\text{-1428}}^{\text{+1562}}$. After eliminating the influence of pseudo-rivers for *N*_Named_ by the ratio of pseudo-rivers ($\text{R}_{\text{P}}$), the *N*_Named_ for China ultimately turns out to be ${\text{3}\text{0}\text{,}\text{201}}_{\text{-1}\text{,}\text{187}}^{\text{+1}\text{,}\text{299}}$.

Even though the main focus here is on rivers in China with catchment area ≥ 100 km^2^, the hybrid stream-order model is capable of estimating numbers of named rivers for varying catchment areas. Supplementary Table 11 lists the extrapolated results for the number of named rivers aggregated by catchment areas obtained from the six typical *P*_f_. In China, the number of named rivers decreases with basin area, whereas the error range related to the number of named rivers increases with catchment area. Low-order rivers which have higher numbers are more likely to lead to an accurate result during random simulation.

**Data sources**

*Bulletin of First National Census for Water* [37]*.* The bulletin separately lists the numbers of rivers with catchment areas ≥ 10,000 km^2^, ≥ 1,000 km^2^, ≥ 100 km^2^, and ≥ 50 km^2^; the values for areas ≥ 100 km^2^ are taken as reference.

*DEMs.* Three DEMs data sets are utilized in this study. First, the drainage networks of China are extracted from the 30 m resolution Aster GDEM (available at <http://reverb.echo.nasa.gov/reverb/)>, which provides the highest resolution data freely available at global scale. These data were launched in the same year as China’s First National Census for Water, and so any disparity caused by a time gap is minimized. Secondly, 90m resolution SRTM DEMs (available at http://www.gscloud.cn/), lunched in 2003, is used to represent the terrain in 2000s. Thirdly, the 900 m resolution GTOPO 30 DEMs (available at <http://earthexplorer.usgs.gov/)>, launched in 1996, is used to represent the terrain in 1990s.

*Vector data of streams and lakes.* Three sets of vector stream and lake data are utilized. National Geomatical Data (available at <http://www.ngcc.cn/>) at 1:4 million resolution provide burned streams in the DEM preprocessing procedure [38]. Hydrosheds Data [3] (available at <http://hydrosheds.cr.usgs.gov>) are used to validate the extracted drainage networks. The Global Lakes and Wetlands Database [2] (GLWD) (available at <http://www.worldwildlife.org>) is used to identify boundaries of sinks when extracting interior rivers.

*Annual mean precipitation of China.* Annual mean precipitation map of China is obtained from the *Atlas of Physical geography of China* [39].

*Aridity Index.* The aridity index [13] is a function of precipitation and potential evapo-transpiration, and is defined as:

*AI= P/PET*^0^ (23)

where *P* is mean annual precipitation; and *PET^0^* is the mean annual potential evapo-transpiration. The aridity index is widely used as an indicator of dry-wet climate. We adopt the climate classification scheme for aridity index values proposed by the Meteorological Standards of China (see Supplementary Information). Two sets of aridity index data are utilized: the annual average aridity index for 1950-2000 (available at <https://perswww.kuleuven.be/~u0055544/aridity/Global_Aridity_PET_Methodolgy.htm>); and the aridity index for June, 2011 (available at http://www.dsac.cn/DataProduct/Detail/201004). The resolution of both data sets is 1 km. The first data set is used in estimating the number of dried-out rivers, and the second is used in identifying which pseudo rivers to exclude.

*Water Occurrence.* The Water Occurrence dataset [25] is accessible via http://global-surface-water.appspot.com/, and presents information on the spatial distribution of surface water at 30 m resolution over the Earth’s surface in the period from 1984 to 2015. The information is given as percentage occurrence frequency, previously treated to ensure temporal consistency.

*Encyclopedia of Rivers and Lakes in China* [34]*.* Information of representative named river networks of China is obtained from *Encyclopedia of Rivers and Lakes in China*. This encyclopedia lists rivers of area ≥ 1,000 km^2^ and provides examples of the traditional approach to naming rivers in China.

*Modified Compound Topographic Index (MCTI).* The *MCTI* is modified from *CTI*, which is a function of slope and upstream flow accumulation, and is defined as:

$\text{CTI}\text{ = ln (} \text{α}/{\tan\text{(π}\text{S}}\text{/180}\text{)}\text{)}$ (24)

where α is the accumulated flow; and $\text{S}$ is the slope (°). *CTI* is obtained from DEMs, and partly reflects the hydrologic characteristics of a basin [40]. However, because both $\alpha$ and $S$ are computed from DEMs, *CTI* does not account for climate conditions, and so is deficient (considering that river networks are products of both climate and geology). Here, we modify traditional *CTI* by replacing $\alpha$ with the annual average aridity index for 1950-2000 which describes the climatic character of a basin. Thus, *MCTI* is defined as:

$\text{MCTI}\text{ = ln (} \text{AI}/{\tan\text{(π}\text{S}\text{/180)}}\text{)}$ (25)

*Population density data.* Two sets of population data are used to calculate population density. Population data for the Qing Dynasty are obtained from the *History of Population in China (Volume 5), Qing Dynasty* [36]. Population data in 2014 are taken from the *China Statistical Yearbook of 2014* [41].

*Data availability*

The data that support the findings of this study are available from the corresponding author upon reasonable request.

**
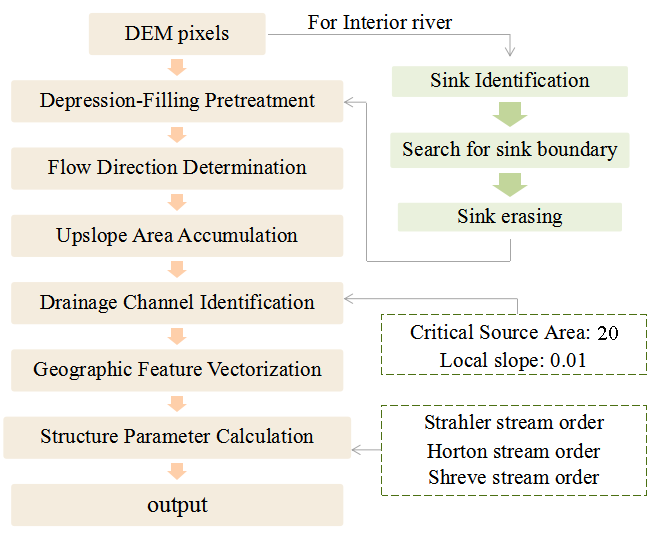
**

Supplementary Fig. 1. Flow chart showing procedure for drainage network extraction.


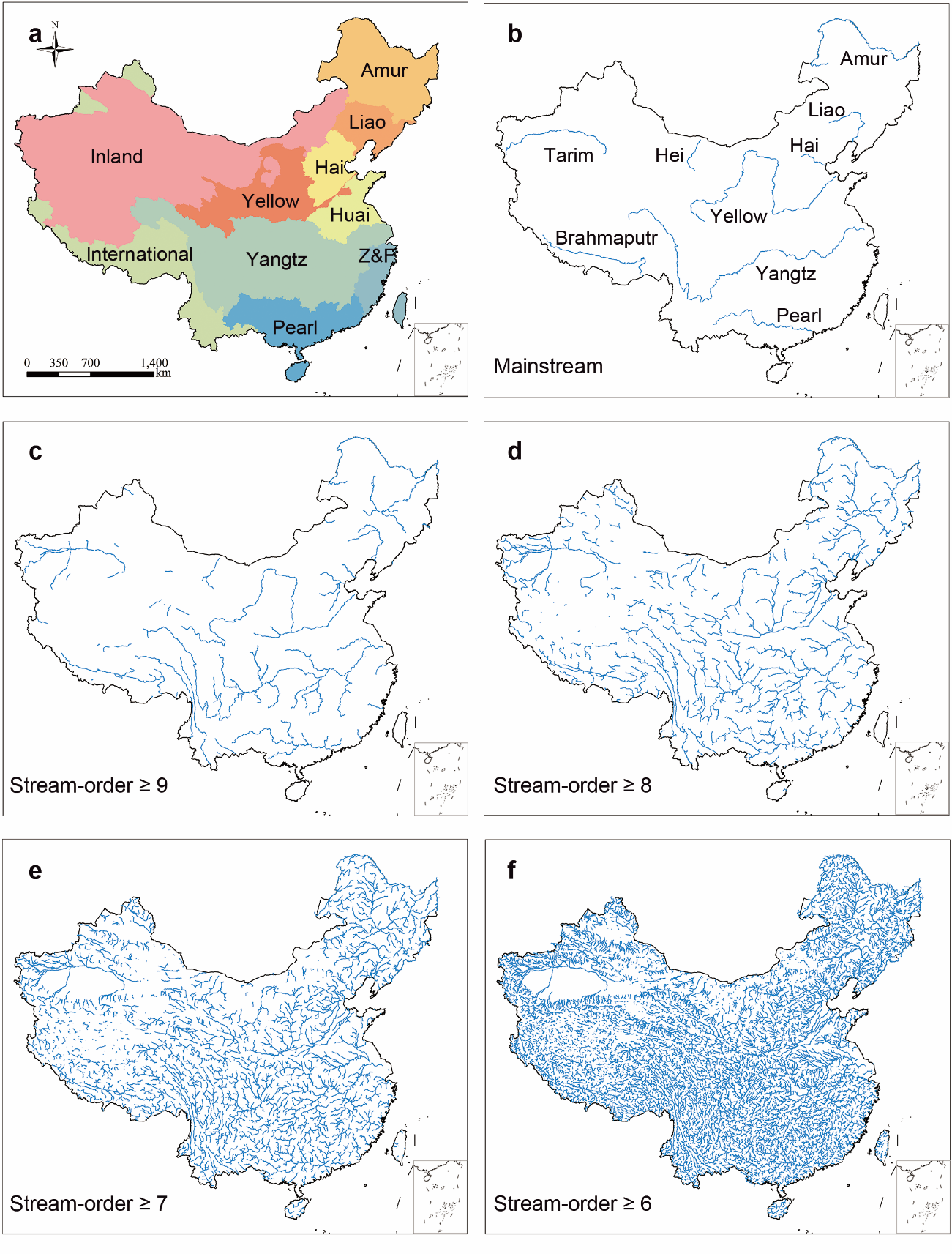


Supplementary Fig. 2. Drainage networks of China. (a) Division into ten basins. (b) Mainstreams of largest rivers. (c) Rivers of stream-order ≥ 9. (d) Rivers of stream-order ≥ 8. (e) Rivers of stream-order ≥ 7. (f) Rivers of stream-order ≥ 6.


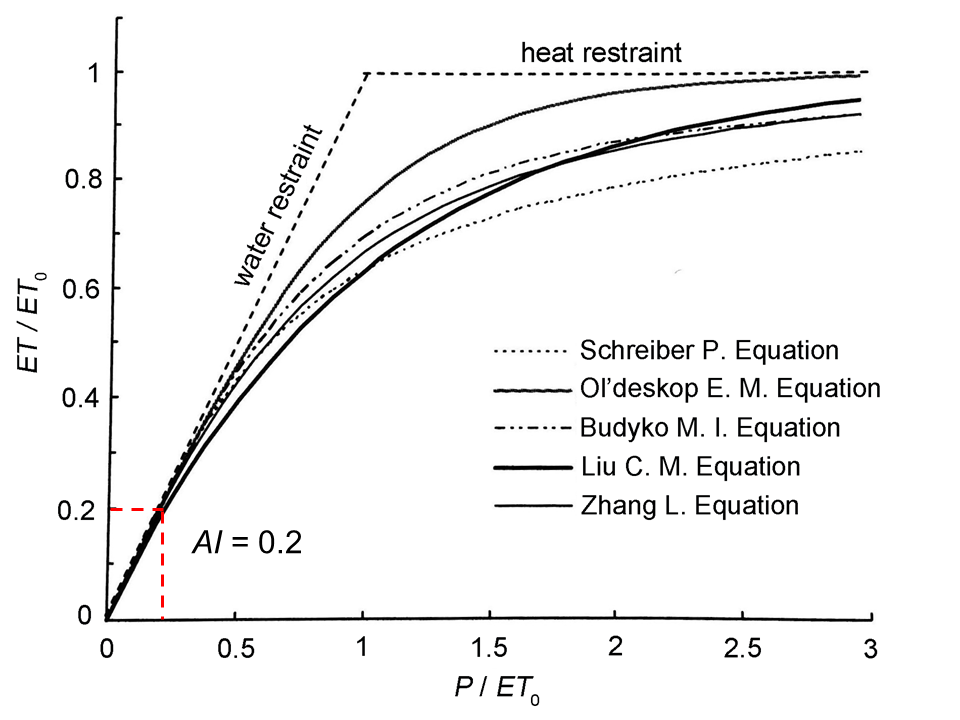


Supplementary Fig. 3. Comparison of different actual evaporation formulas (revised from Liu, 2014 [42]).





Supplementary Fig. 4. Distribution of water-erosion area and no-water erosion area in China.


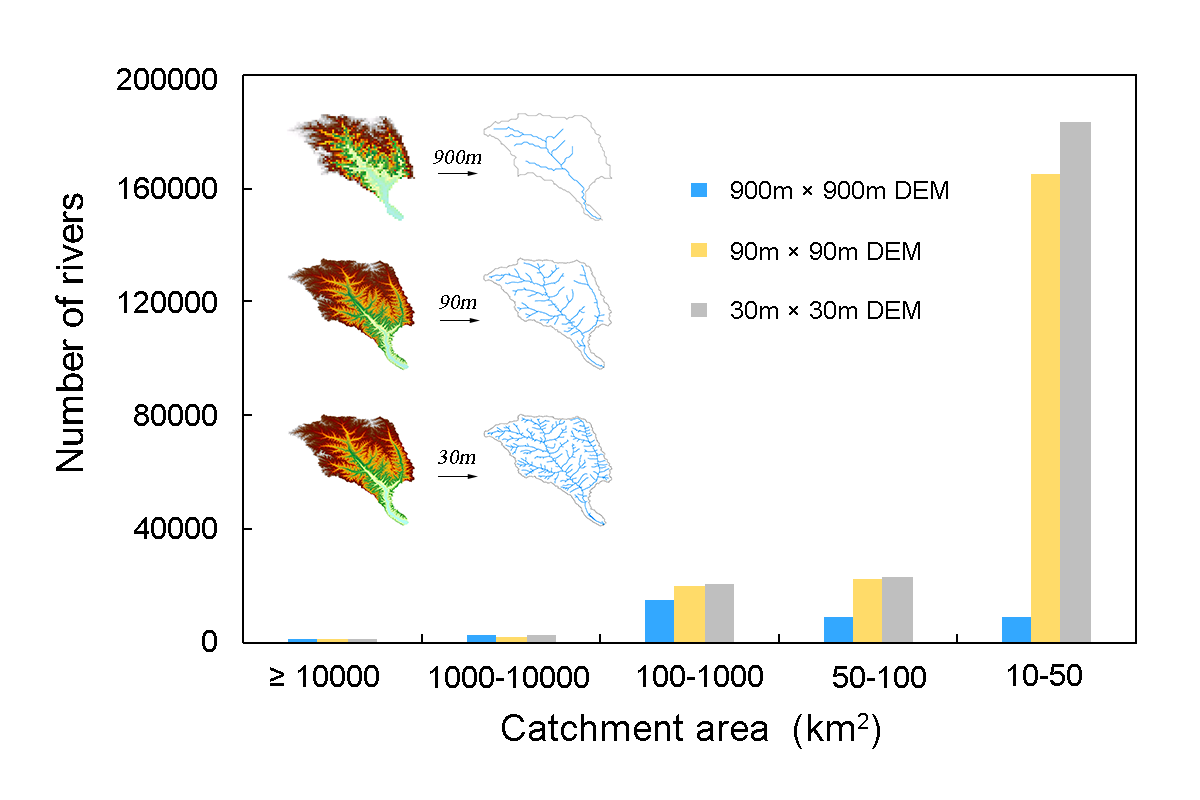


Supplementary Fig. 5. Numbers of rivers with different catchment areas extracted from different DEMs.


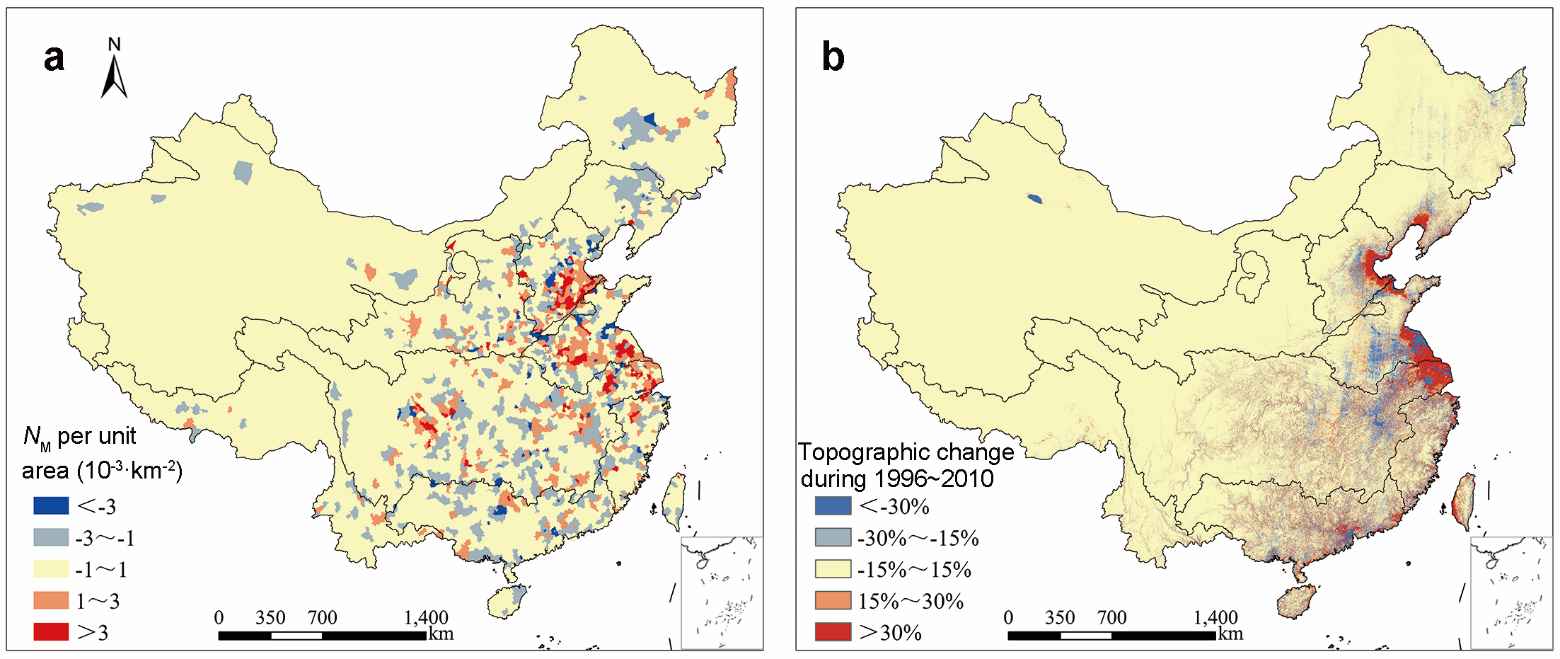


Supplementary Fig. 6. Distributions across China of: (a) *N*_M_ per unit area. (b) Relative difference of topographic change in terms of elevation values during 1996–2010.


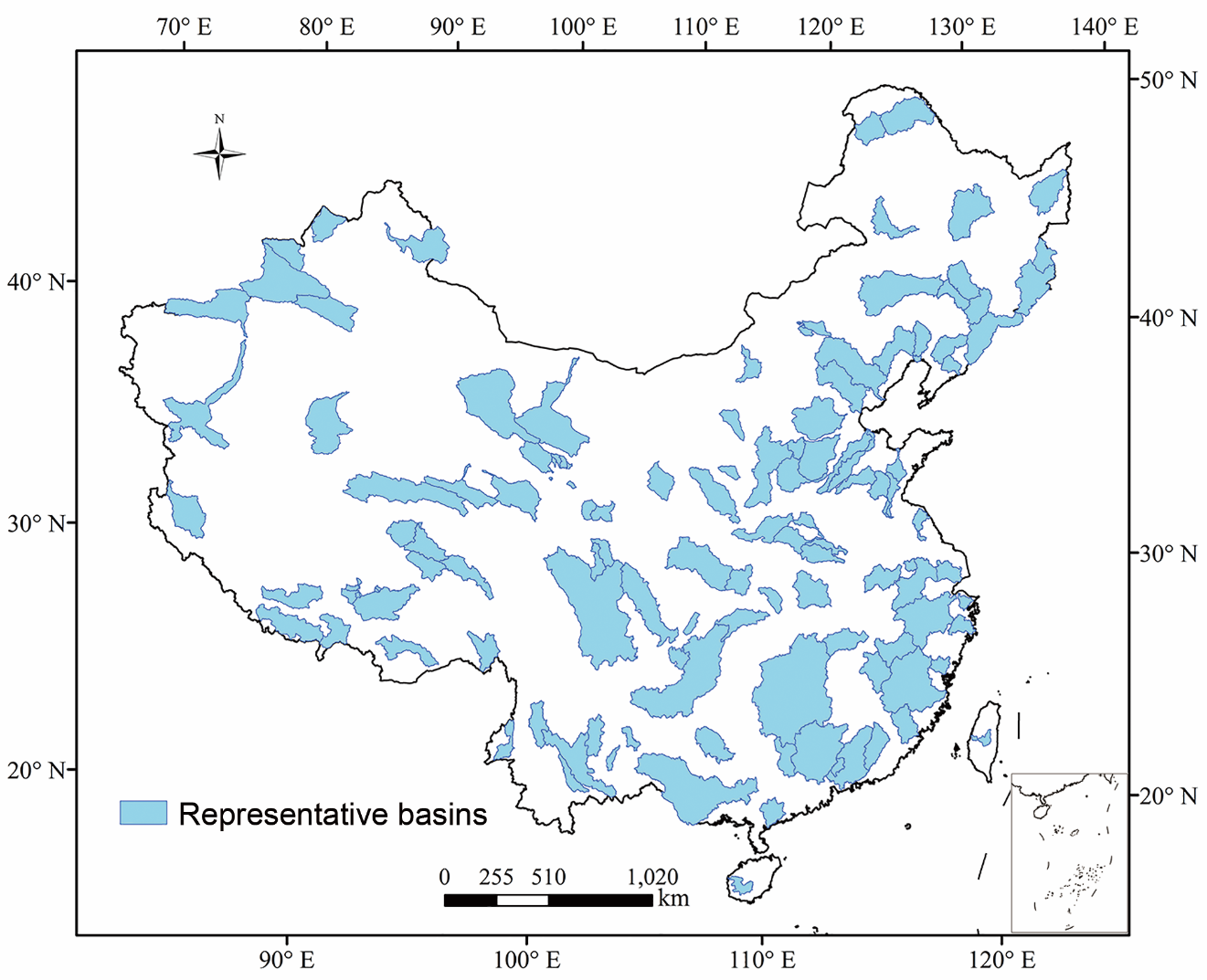


Supplementary Fig. 7. Distribution of 107 representative basins throughout China, taken from *Encyclopedia of Rivers and Lakes in China*.


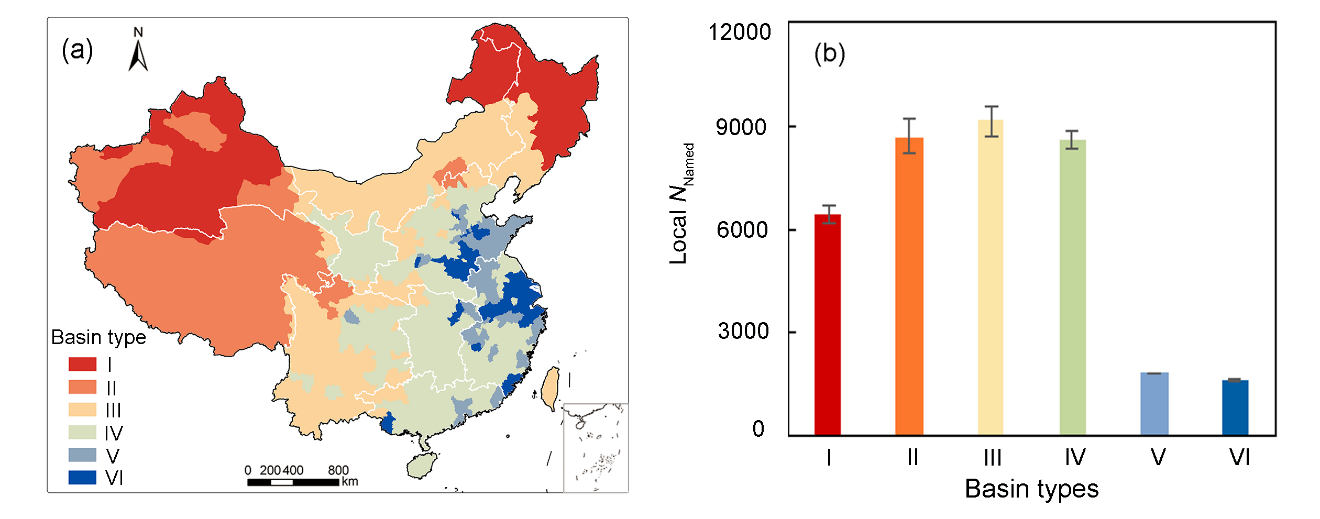


Supplementary Fig. 8. Estimated *N*_Named_ in China: (a) Distribution of six types of basins in China with different population densities. (b) Histogram displaying local *N*_Named_ using corresponding proportions of the three stream-ordering schemes.

Supplementary Table 1.

Numbers of rivers ≥ 100 km^2^ in China obtained from National Census and different stream-ordering methods, numbers of rivers after excluding pseudo-rivers, rivers extrapolated from low-resolution topographic data, difference in number of rivers due to topographic change, and numbers of named rivers.

| Basin | $\text{N}_{\text{Census}}$ | $\text{N}_{\text{Horton}}$ | $\text{N}_{\text{Stahler}}$ | $\text{N}_{\text{Shreve}}$ | ${\text{N}\text{'}}_{\text{Horton}}$ | ${\text{N}\text{'}}_{\text{Strahler}}$ | ${\text{N}\text{'}}_{\text{Shreve}}$ | $\text{N}_{\text{T}}$ | $\text{N}_{\text{M}}$ | $\text{N}_{\text{Named}}$ |
| --- | --- | --- | --- | --- | --- | --- | --- | --- | --- | --- |
| Yangtze | 5,276 | 5,194 | 7,246 | 125,518 | 5,194 | 6,026 | 104,387 | 4,714 | 419 | 30,201 |
| Yellow | 2,061 | 2,253 | 3,096 | 72,532 | 1,809 | 2,575 | 60,321 | 1,963 | 130 |  |
| Amur | 2,428 | 2,737 | 3,839 | 60,266 | 2,676 | 3,193 | 50,120 | 4,577 | 259 |  |
| Liao | 791 | 903 | 1,298 | 19,184 | 809 | 1,079 | 15,954 | 1,439 | 123 |  |
| Hai | 892 | 949 | 1,340 | 28,060 | 908 | 1,114 | 23,336 | 1,700 | -13 |  |
| Huai | 1,266 | 993 | 1,405 | 20,011 | 993 | 1,168 | 16,642 | 1,308 | -42 |  |
| Fujian & Zhejiang | 694 | 701 | 993 | 22,378 | 701 | 826 | 18,611 | 784 | 74 |  |
| Pearl | 1,685 | 1,723 | 2,413 | 47,092 | 1,723 | 2,007 | 39,164 | 1,831 | 259 |  |
| International | 2,467 | 2,834 | 3,961 | 123,858 | 2,488 | 3,294 | 103,007 | 3,009 | 148 |  |
| Interior | 5,349 | 8,900 | 12,424 | 151,047 | 5,309 | 10,332 | 125,618 | 6,930 | 596 |  |
| ∑ | 22,909 | 27,187 | 38,015 | 669,946 | 22,610 | 31,615 | 557,161 | 28,254 | 1,953 |  |

The minus sign means the number of rivers increases due to topographic change.

$\text{N}_{\text{Name}\text{d}}$: the number of named rivers in China estimated calculated using different $P_{f}$ in different regions.

Supplementary Table 2.

Numbers of rivers (and pseudo-rivers) ≥ 100 km^2^ in China based on Horton scheme within different catchment areas for the ten basins of China using 30 m × 30 m DEMs.

| Basin | Catchment Area (km^2^) | | | | | |
| --- | --- | --- | --- | --- | --- | --- |
|  | ≥100,000 | 10,000–100,000 | 1,000–10,000 | 100–1,000 | 50–100 | 10–50 |
| Yangtze | 8 | 41 | 450 | 4,695 | 5,193 | 39,962 |
| Yellow | 2 | 16 | 190 | 2,045 | 2,280 | 16,204 |
| Amur | 2 | 33 | 235 | 2,467 | 2,849 | 20,724 |
| Liao | 1 | 11 | 85 | 806 | 812 | 5,496 |
| Hai | 2 | 12 | 80 | 855 | 940 | 7,369 |
| Huai | 2 | 7 | 100 | 884 | 1,003 | 7,593 |
| Fujian & Zhejiang | 0 | 7 | 57 | 637 | 622 | 4,970 |
| Pearl | 1 | 13 | 162 | 1,547 | 1,706 | 13,132 |
| International | 3 | 23 | 246 | 2,562 | 2,915 | 22,065 |
| Interior | 2 | 55 | 789 | 8,054 | 8,806 | 77,870 |
| ∑ | 23 | 218 | 2,394 | 24,552 | 27,126 | 215,385 |

Supplementary Table 3.

Numbers of pseudo-rivers within a given catchment area for ten basins of China.

| Basin | Catchment Area (km^2^) | | |
| --- | --- | --- | --- |
|  | 100–1000 | 50–100 | 10–50 |
| Yangtze | 0 | 0 | 0 |
| Yellow | 444 | 952 | 10,530 |
| Amur | 61 | 70 | 528 |
| Liao | 94 | 88 | 568 |
| Hai | 41 | 63 | 355 |
| Huai | 0 | 1 | 0 |
| Fujian & Zhejiang | 0 | 0 | 0 |
| Pearl | 0 | 0 | 0 |
| International | 346 | 857 | 12,118 |
| Interior | 3,591 | 5,110 | 45,110 |
| ∑ | 4,577 | 7,141 | 69,209 |

Supplementary Table 4.

Numbers of rivers in China with different catchment area extracted from 90m ×90m DEMs.

| Basin | Catchment Area (km^2^) | | | | | |
| --- | --- | --- | --- | --- | --- | --- |
|  | ≥100,000 | 10,000–100,000 | 1,000–10,000 | 100–1,000 | 50–100 | 10–50 |
| Yangtze | 6 | 39 | 459 | 4624 | 5405 | 39008 |
| Yellow | 2 | 18 | 195 | 2137 | 2313 | 17640 |
| Amur | 2 | 35 | 260 | 2742 | 3076 | 21537 |
| Liao | 1 | 10 | 74 | 744 | 777 | 6101 |
| Hai | 2 | 10 | 82 | 822 | 909 | 7336 |
| Huai | 2 | 6 | 100 | 774 | 906 | 7291 |
| Fujian & Zhejiang | 0 | 5 | 44 | 469 | 456 | 3698 |
| Pearl | 1 | 12 | 150 | 1435 | 1595 | 12548 |
| International | 3 | 20 | 230 | 2334 | 2711 | 20383 |
| Interior | 1 | 50 | 692 | 7084 | 7865 | 58823 |
| ∑ | 20 | 205 | 2,286 | 23,165 | 26,013 | 194,365 |

Supplementary Table 5.

Numbers of rivers in China with different catchment area extracted from 900m ×900 m DEMs.

| Basin | Catchment Area (km^2^) | | | | | |
| --- | --- | --- | --- | --- | --- | --- |
|  | ≥100,000 | 10,000–100,000 | 1,000–10,000 | 100–1,000 | 50–100 | 10–50 |
| Yangtze | 8 | 36 | 469 | 3,020 | 1,777 | 1,371 |
| Yellow | 2 | 15 | 190 | 987 | 214 | 32 |
| Amur | 2 | 35 | 245 | 2,215 | 1,580 | 1,652 |
| Liao | 1 | 11 | 80 | 687 | 495 | 483 |
| Hai | 1 | 13 | 67 | 551 | 391 | 409 |
| Huai | 1 | 10 | 81 | 483 | 342 | 281 |
| Fujian & Zhejiang | 0 | 6 | 58 | 430 | 222 | 162 |
| Pearl | 1 | 14 | 166 | 992 | 545 | 375 |
| International | 3 | 23 | 256 | 1,532 | 941 | 819 |
| Interior | 2 | 53 | 742 | 6,327 | 4,084 | 4,876 |
| ∑ | 21 | 216 | 2,354 | 17,224 | 10,591 | 10,460 |

Supplementary Table 6.

Numbers of rivers with different catchment areas extrapolated from 900 m × 900 m DEMs

| Basin | *N*_900(100-1000)_ | *N*_900(50-100)_ | *N*_900(10-50)_ | $\text{N}_{\text{T}}$ | $\text{N}_{\text{Tmin}}$ | $\text{N}_{\text{Tmax}}$ |
| --- | --- | --- | --- | --- | --- | --- |
| Yangtze | 5112 | 5623 | 50099 | 5625 | 2952 | 7079 |
| Yellow | 2071 | 2278 | 20296 | 2278 | 1195 | 2867 |
| Amur | 2671 | 2938 | 26171 | 2953 | 1556 | 3712 |
| Liao | 872 | 959 | 8546 | 964 | 508 | 1212 |
| Hai | 730 | 803 | 7157 | 811 | 429 | 1019 |
| Huai | 883 | 971 | 8652 | 975 | 513 | 1226 |
| Fujian & Zhejiang | 632 | 695 | 6196 | 696 | 366 | 876 |
| Pearl | 1809 | 1990 | 17732 | 1990 | 1044 | 2505 |
| International | 2790 | 3069 | 27346 | 3072 | 1613 | 3866 |
| Interior | 8088 | 8897 | 79260 | 8885 | 4655 | 11185 |
| ∑ | 25659 | 28224 | 251454 | 28250 | 14832 | 35547 |

Supplementary Table 7.

Numbers of rivers in China with different catchment area extracted from resampled 900m ×900 m DEMs.

| Basin | Catchment Area (km^2^) | | | | | |
| --- | --- | --- | --- | --- | --- | --- |
|  | ≥100,000 | 10,000–100,000 | 1,000–10,000 | 100–1,000 | 50–100 | 10–50 |
| Yangtze | 8 | 42 | 464 | 2748 | 1523 | 1112 |
| Yellow | 2 | 15 | 177 | 933 | 208 | 21 |
| Amur | 2 | 33 | 249 | 1994 | 1382 | 1183 |
| Liao | 1 | 10 | 79 | 593 | 382 | 352 |
| Hai | 1 | 10 | 77 | 553 | 371 | 349 |
| Huai | 1 | 6 | 107 | 486 | 300 | 178 |
| Fujian & Zhejiang | 0 | 6 | 56 | 383 | 185 | 91 |
| Pearl | 1 | 15 | 163 | 833 | 446 | 313 |
| International | 3 | 22 | 254 | 1443 | 883 | 778 |
| Interior | 1 | 58 | 724 | 5887 | 4095 | 4578 |
| ∑ | 20 | 217 | 2350 | 15853 | 9775 | 8955 |

Supplementary Table 8.

Numbers of rivers ≥ 100 km^2^ in ten basins in China related to changes in topography.

| Basin | *N*_30_ | *N*_900_ | *N*_900_' | *N*_900_*-* *N*_900_' | *N*_30_ - *N*_900_' | *P*_M_ | *N*_M_ | *N*_Mmin_ | *N*_Mmax_ | Slope(º) |
| --- | --- | --- | --- | --- | --- | --- | --- | --- | --- | --- |
| Yangtze | 5,194 | 3,533 | 3,262 | 271 | 1,932 | 7.67% | 419 | 187 | 536 | 13.4 |
| Yellow | 2,253 | 1,194 | 1,127 | 67 | 1,126 | 5.61% | 130 | 18 | 222 | 9.3 |
| Amur | 2,737 | 2,497 | 2,278 | 219 | 459 | 8.77% | 259 | 199 | 282 | 4.6 |
| Liao | 903 | 779 | 683 | 96 | 220 | 12.32% | 123 | 86 | 126 | 5.4 |
| Hai | 949 | 632 | 641 | -9 | 308 | -1.42% | -13 | -22 | 33 | 6.32 |
| Huai | 993 | 575 | 600 | -25 | 393 | -4.35% | -42 | -42 | 29 | 2.1 |
| Fujian & Zhejiang | 701 | 494 | 445 | 49 | 256 | 9.92% | 74 | 38 | 84 | 14.2 |
| Pearl | 1,723 | 1,173 | 1,012 | 161 | 711 | 13.73% | 259 | 130 | 259 | 11.1 |
| International | 2,834 | 1,814 | 1,722 | 92 | 1,112 | 5.07% | 148 | 44 | 245 | 18.1 |
| Interior | 8,900 | 7,124 | 6,670 | 454 | 2,230 | 6.37% | 596 | 357 | 760 | 5.8 |
| ∑ | 27,187 | 19,815 | 18,440 | 1,375 | 8,747 | 6.94% | 1,953 | 995 | 2,576 | - |

*P*_M_ is the percentage of migrated rivers defined as the ratio of (*N*_900_*-* *N*_900'_) and *N*_900._

Supplementary Table 9.

Scenarios, listing proportions of the three stream-ordering schemes.

| $\text{NP}_{\text{f}}$ | *P*_HO_ | *P*_ST_ | *P*_SH_ | *N*_Mixed_ |
| --- | --- | --- | --- | --- |
| 1 | 1 | 0 | 0 | 27,143 |
| 2 | 0.9 | 0 | 0.1 | 27,209 |
| 3 | 0.9 | 0.1 | 0 | 27,225 |
| 4 | 0.8 | 0 | 0.2 | 27,240 |
| 5 | 0.8 | 0.1 | 0.1 | 27,284 |
| 6 | 0.7 | 0 | 0.3 | 27,284 |
| 7 | 0.8 | 0.2 | 0 | 27,331 |
| 8 | 0.6 | 0 | 0.4 | 27,368 |
| 9 | 0.7 | 0.1 | 0.2 | 27,379 |
| 10 | 0.7 | 0.2 | 0.1 | 27,476 |
| 11 | 0.5 | 0 | 0.5 | 27,522 |
| 12 | 0.6 | 0.1 | 0.3 | 27,553 |
| 13 | 0.7 | 0.3 | 0 | 27,600 |
| 14 | 0.6 | 0.2 | 0.2 | 27,720 |
| 15 | 0.5 | 0.1 | 0.4 | 27,857 |
| 16 | 0.4 | 0 | 0.6 | 27,876 |
| 17 | 0.6 | 0.3 | 0.1 | 27,919 |
| 18 | 0.6 | 0.4 | 0 | 28,134 |
| 19 | 0.5 | 0.2 | 0.3 | 28,193 |
| 20 | 0.5 | 0.3 | 0.2 | 28,510 |
| 21 | 0.4 | 0.1 | 0.5 | 28,565 |
| 22 | 0.5 | 0.4 | 0.1 | 28,749 |
| 23 | 0.3 | 0 | 0.7 | 28,880 |
| 24 | 0.5 | 0.5 | 0 | 29,047 |
| 25 | 0.4 | 0.2 | 0.4 | 29,107 |
| 26 | 0.4 | 0.3 | 0.3 | 29,532 |
| 27 | 0.4 | 0.4 | 0.2 | 29,923 |
| 28 | 0.4 | 0.5 | 0.1 | 30,171 |
| 29 | 0.3 | 0.1 | 0.6 | 30,200 |
| 30 | 0.4 | 0.6 | 0 | 30,434 |
| 31 | 0.3 | 0.2 | 0.5 | 31,102 |
| 32 | 0.3 | 0.3 | 0.4 | 31,643 |
| 33 | 0.3 | 0.4 | 0.3 | 32,047 |
| 34 | 0.3 | 0.5 | 0.2 | 32,232 |
| 35 | 0.3 | 0.6 | 0.1 | 32,327 |
| 36 | 0.3 | 0.7 | 0 | 32,378 |
| 37 | 0.2 | 0 | 0.8 | 32,496 |
| 38 | 0.2 | 0.8 | 0 | 34,777 |
| 39 | 0.2 | 0.1 | 0.7 | 34,950 |
| 40 | 0.2 | 0.7 | 0.1 | 35,146 |

Supplementary Table 9.

Scenarios, listing proportions of the three stream-ordering schemes. *(Continued)*

| $\text{NP}_{\text{f}}$ | *P*_HO_ | *P*_ST_ | *P*_SH_ | *N*_Mixed_ |
| --- | --- | --- | --- | --- |
| 41 | 0.2 | 0.6 | 0.2 | 35,513 |
| 42 | 0.2 | 0.5 | 0.3 | 35,830 |
| 43 | 0.2 | 0.2 | 0.6 | 35,968 |
| 44 | 0.2 | 0.4 | 0.4 | 36,119 |
| 45 | 0.2 | 0.3 | 0.5 | 36,307 |
| 46 | 0.1 | 0.9 | 0 | 37,102 |
| 47 | 0 | 1 | 0 | 38,015 |
| 48 | 0.1 | 0.8 | 0.1 | 38,057 |
| 49 | 0.1 | 0.7 | 0.2 | 39,350 |
| 50 | 0 | 0.9 | 0.1 | 39,911 |
| 51 | 0.1 | 0.6 | 0.3 | 40,790 |
| 52 | 0 | 0.8 | 0.2 | 42,316 |
| 53 | 0.1 | 0.5 | 0.4 | 42,625 |
| 54 | 0.1 | 0.4 | 0.5 | 44,718 |
| 55 | 0 | 0.7 | 0.3 | 45,364 |
| 56 | 0.1 | 0.3 | 0.6 | 47,363 |
| 57 | 0 | 0.6 | 0.4 | 49,446 |
| 58 | 0.1 | 0.2 | 0.7 | 50,427 |
| 59 | 0.1 | 0.1 | 0.8 | 53,341 |
| 60 | 0.1 | 0 | 0.9 | 53,520 |
| 61 | 0 | 0.5 | 0.5 | 55,132 |
| 62 | 0 | 0.4 | 0.6 | 63,499 |
| 63 | 0 | 0.3 | 0.7 | 77,460 |
| 64 | 0 | 0.2 | 0.8 | 103,854 |
| 65 | 0 | 0.1 | 0.9 | 171,600 |
| 66 | 0 | 0 | 1 | 669,946 |

Supplementary Table 10.

Information on four human aggregation modes of basins in China in terms of population density.

| Basin types | Population density in Qing Dynasty (person · km^-2^) | Range of $\text{NP}_{\text{f}}$ | Maximum occurrence $\text{NP}_{\text{f}}$ | Proportions of the three schemes | Frequency  (*A*/*B*)* |
| --- | --- | --- | --- | --- | --- |
| I | < 0.4 | 24–52 |  | 0.4:0.6:0 | 4/13 |
| II | 0.4–2 | 24–48 |  | 0.3:0.6:0.1 | 4/24 |
| III | 2–30 | 28–61 |  | 0.2:0.7:0.1 | 4/22 |
| IV | 30–140 | 27–48 |  | 0.1:0.8:0.1 | 11/30 |
| V | 140–200 | 36–52 |  | 0:1:0 | 3/12 |
| VI | > 200 | 48–52 |  | 0:0.9:0.1 | 2/6 |

*A* is the number of basins with most frequent occurrences of $\text{NP}_{\text{f}}$; *B* is the number of basins located in each basin type.

Supplementary Table 11.

Number of named rivers in China aggregated by catchment area.

| Basin Area (km^2^) | $\text{N}_{\text{Named}}$ | $\text{N}_{\text{Named}\text{-min}}$ | $\text{N}_{\text{Named}\text{-max}}$ |
| --- | --- | --- | --- |
| ≥ 10000 | 457 | 256 | 735 |
| 1000–10000 | 2,917 | 2,225 | 3,719 |
| 100–1000 | 26,945 | 24,264 | 29,755 |
| 50–100 | 29,501 | 26,682 | 32,424 |
| 10–50 | 220,578 | 212,168 | 228,977 |

References

1. Bai, R, Li, TJ and Huang, YF *et al.* An efficient and comprehensive method for drainage network extraction from DEM with billions of pixels using a size-balanced binary search tree. *Geomorphology* 2015; **238**: 56–67.
2. Lehner, B and Döll, P. Development and validation of a global database of lakes, reservoirs and wetlands. *J. Hydrol.* 2004; **296**: 1–22.
3. Lehner, B, Verdin, K and Jarvis, A. New global hydrography derived from spaceborne elevation data. *Eos. Transactions.* 2008; **89**: 93–94.
4. Horton, RE. Erosional development of streams and their drainage basins: hydro-physical approach to quantitative morphology. *Geol. Soc. Am. Bull.* 1945; **56**: 275–370.
5. Leading group office of the first national census for water. *General Plan for the First National Census for Water (in Chinese).* Beijing: China Water &Power Press, 2010.
6. Strahler, AN. Hypsometric (area-altitude) analysis of erosional topography. *Geol. Soc. Am. Bull.* 1952; **63**: 1117–1142.
7. Strahler, AN. Quantitative analysis of watershed geomorphology. *EOS Trans. AGU* 1957; **38**: 913–920.
8. Shreve, R. Statistical Law of Stream Numbers. *J. Geol.* 1966; **74**: 17–37.
9. O’Callaghan, JF and Mark, DM. The extraction of drainage networks from digital elevation data. *Computer Vision, Graphics, and Image Processing* 1984; **28**: 323–344.
10. Schneider, A, Jost, A and Coulon, C *et al.* Global-scale river network extraction based on high-resolution topography and constrained by lithology, climate, slope, and observed drainage density. *Geophys. Res. Lett.* 2017; **44**: 2773–2781.
11. Sangireddy, H, Carothers, RA and Stark, CP *et al.* Controls of climate, topography, vegetation, and lithology on drainage density extracted from high resolution topography data. *J. Hydrol.* 2016; **537**: 271–282.
12. Rui, XF. *Principles of Hydrology (in Chinese).* Beijing: China Water & Power Press, 2004.
13. Middleton, N and Thomas, DS and UNEP. *World Atlas of Desertification.* London: Edward Arnold, 1992.
14. Mao, F, Sun, H and Yang, HL. Research Progress in Dry/wet Climate Zoning (in Chinese). *Progress in Geography* 2011; **30**: 17–26.
15. Budyko, MI. *Evaporation under Natural Conditions.* Jerusalem: Isr. Program for Sci. Transl, 1948.
16. Department of Hydrology and Water Resources, University of Arizona. *The Quantitative Features of China’s Water Resources: An Overview.* Tucson, AZ: Department of Hydrology and Water Resources, University of Arizona, 1983. (Report on Natural Resource Systems No. 38).
17. Ol’deskop, EM. *On Evaporation from the Surface of River Basins (in Russia).* Tartu: Tartu Univ, 1911.
18. Zhang, L, Dawes, W and Walker, GR. Response of mean annual evapotranspiration to vegetation changes at catchment scale. *Water Resour. Res.* 2001; **37**: 701–708.
19. Tang, KL. *Soil and Water Conservation in China (in Chinese)*. Beijing: Science Press, 2004*.*
20. Zhu, TX. Gully and tunnel erosion in the hilly Loess Plateau region, China. *Geomorphology* 2012; **153**: 144–155.
21. Sorg, A, Bolch, T and Stoffel, M. Climate change impacts on glaciers and runoff in Tien Shan (Central Asia). *Nat. Clim. Chang.* 2012; **2**: 725–731.
22. Xiao, J, Jin, ZD and Wang, J *et al.* Hydrochemical characteristics, controlling factors and solute sources of groundwater within the Tarim River Basin in the extreme arid region, NW Tibetan Plateau. *Quat. Int.* 2015; **380**: 237–246.
23. Lutz, AF, Immerzeel, WW and Shrestha, AB *et al.* Consistent increase in High Asia's runoff due to increasing glacier melt and precipitation. *Nat. Clim. Chang*. 2014; **4**: 587–592.
24. Li, Z, Lin, XQ and Coles, AE *et al.* Catchment-scale surface water-groundwater connectivity on china's loess plateau. *Catena* 2017; **152**: 268–276.
25. Pekel, JF, Cottam, A and Gorelick, N *et al.* High-resolution mapping of global surface water and its long-term changes. *Nature* 2016; **540**: 418–422.
26. Widder, S, Besemer, K and Singer, GA *et al.* Fluvial network organization imprints on microbial co-occurrence networks. *Proc. Natl. Acad. Sci. U. S. A.* 2014; **111**: 12799–12804.
27. Ni, JR and Ma, AN. *River dynamic geomorphology (in Chines).* Beijing: Peking University Press, 1998.
28. Zanardo, S, Zaliapin, I and Foufoula-Georgiou, E. Are American rivers Tokunaga self‐similar? New results on fluvial network topology and its climatic dependence. *JGR* 2013; **118**: 166–183.
29. Yang, DW, Herath, S and Musiake, K. Spatial resolution sensitivity of catchment geomorphologic properties and the effect on hydrological simulation. *Hydrol. Process.* 2001; **15**: 2085–2099.
30. Yang, L, Xu, YP and Han, LF et al. River networks system changes and its impact on storage and flood control capacity under rapid urbanization. Hydrol. Process. 2016; 30: 2401–2412.
31. Zheng, HB. Birth of the Yangtze River: age and tectonic-geomorphic implications. Natl. Sci. Rev. 2015; 2: 438–453.
32. Deng, XZ, Huang, JK and Rozelle, S. *et al.* Impact of urbanization on cultivated land changes in China. *Land Use Pol.* 2015; **45**: 1–7.
33. Meierdiercks, KL, Smith, JA and Baeck, ML *et al.* Analyses of urban drainage network structure and its impact on hydrologic response. *J. Am. Water Resour. Assoc.* 2010; **46**: 932–943.
34. Editorial Committee of Encyclopedia of Rivers and Lakes in China. *Encyclopedia of Rivers and Lakes in China (in Chinese)*. Beijing: China Water Power Press, 2014.
35. Roberts, GO and Rosenthal, JS. Coupling and ergodicity of adaptive Markov chain Monte Carlo algorithms. *J. Appl. Probab.* 2007; **44**: 458–475.
36. Ge, JX. *History of Population in China (Volume 5), Qing Dynasty (in Chinese).* Shanghai: Fudan University Press, 2001.
37. Ministry of Water Resources, PRC and National Bureau of Statistics, PRC. *Bulletin of First National Census for Water.* Beijing: China Water &Power Press, 2013.
38. Callow, JN, Van Niel, KP and Boggs, GS. How does modifying a DEM to reflect known hydrology affect subsequent terrain analysis? *J. Hydrol.* 2007; **332**: 30–39.
39. Department of Geography, Northwest Normal Universitys. *Atlas of Physical geography of China.* Beijing: SinoMaps Press, 1984.
40. Moore, ID, Grayson, RB and Ladson, AR. Digital terrain modelling: A review of hydrological, geomorphological, and biological applications. *Hydrol. Process.* 1991; **5:** 3–30.
41. National Bureau of Statistics of China. *China Statistical Yearbook 2014* *(in Chinese).* Beijing: China Statistics Press, 2014.
42. Liu, CM. *Hydrogeography of China (in Chinese).* Beijing: Science Press, 2014.

Complete Abbreviation List

*AI*: Aridity Index.

$\text{D}_{\text{P}}$: Number of pseudo-rivers with catchment area ≥ 100 km^2^ per unit area, 10^-3^·km^-2^.

$\text{D}_{\text{Horton}}$: Number of rivers estimated by Horton stream-ordering scheme per unit area, 10^-3^ km^-2^.

*Dif*: Relative difference between $\text{n}_{\text{Mixed}}$ and $\text{n}_{\text{Named}}$for representative basins.

$\text{D}_{\text{M}}$: Change in number of rivers ≥ 100 km^2^ due to topographic change per unit area, 10^-3^·km^-2^.

$\text{D}_{\text{Named}}$: Number of named rivers estimated by hybrid stream-order rule per unit area, 10^-3^·km^-2^.

$\text{D}_{\text{1776}}$: Population density in Early Qing Dynasty (1776), person·km^-2^.

*f(x)*: Probability density function.

*I(x)*: Integral function of *f(x).*

*ID*_Shreve_, *ID*_Strahler_, and *ID*_Horton_: Value of *ID* for each elementary reach derived from overlay of the three river networks.

*MCTI*: modified compound topographic index, a function of surface flow accumulation, aridity, and topographic slope.

*n*: Stream order.

$\text{n}_{\text{d}}$: Stream order of downstream river reach.

*n*_HO_: Total number of reaches consistent with Horton scheme in a representative basin.

*n*_Horton_: Number of rivers estimated by Horton stream-ordering scheme in a representative basin.

$\text{n}_{\text{M}}$: Actual number of river reaches,$\text{n}_{\text{M}}\text{=}\lim_{\text{M→∞}} \overline{\text{N}_{\text{MC}}}$.

$\text{n}_{\text{max}}$: Maximum stream order.

*n*_SH_: Total number of reaches consistent with Shreve scheme in a representative basin.

*n*_Shreve_: Number of rivers estimated by Shreve stream-ordering scheme in a representative basin.

$\text{n}_{\text{Mixed}}$: Number of rivers obtained from random naming simulation based on Monte Carlo method for representative basins.

*n*_ST_: Total number of reaches consistent with Strahler scheme in a representative basin.

*n*_Strahler_: Number of rivers estimated by Strahler stream-ordering scheme in a representative basin.

*n*_Total_: Total number of elemental reaches in a representative basin.

$\text{n}_{\text{Named}}$: Number of historic named rivers for representative basins.

*n*_1,_ *n*_2_: Respective stream orders of upstream rivers at the bifurcation.

$\text{N}_{\text{Census}}$: Number of rivers with catchment area ≥ 100 km^2^ reported in National Census.

$\text{N}_{\text{Horton}}$: Number of rivers with catchment area ≥ 100 km^2^ estimated by Horton stream-ordering scheme.

$\text{N}_{\text{Horton}}\text{'}$: Number of rivers with catchment area ≥ 100 km^2^ estimated by Horton stream-ordering scheme after exclusion of pseudo-rivers.

$\text{N}_{\text{M}}$: Discrepancy in number of rivers with catchment area ≥ 100 km^2^ caused by changes in land topography from 1996 and 2011.

$\text{N}_{\text{Mixed}}$: Number of rivers (catchment area ≥ 100 km^2^) estimated by hybrid stream-order rule.

$\text{N}_{\text{Mmin}}$, $\text{N}_{\text{Mmax}}$: Minimum and maximum values of $\text{N}_{\text{M}}$.

$\text{N}_{\text{MC}}$: Total number of river reaches obtained from Monte Carlo simulation.

$\overline{\text{N}_{\text{MC}}}$: mean value of $\text{N}_{\text{MC}}$

$\text{N}_{\text{Named}}$: Number of named rivers in 1990s with catchment area ≥ 100 km^2^.

$\text{N}_{\text{P}}$: Number of pseudo-rivers with catchment area ≥ 100 km^2^.

$\text{NP}_{\text{f}}$ : Scenario number related to $\text{P}_{\text{f}}$.

$\text{N}_{\text{Shreve}}$: Number of rivers with catchment area ≥ 100 km^2^ estimated by Shreve stream-ordering scheme.

$\text{N}_{\text{Shreve}}\text{'}$: Number of rivers with catchment area ≥ 100 km^2^ estimated by Shreve stream-ordering scheme after correction of the errors induced by pseudo-rivers.

$\text{N}_{\text{Strahler}}$: Number of rivers with catchment area ≥ 100 km^2^ estimated by Strahler stream-ordering scheme.

$\text{N}_{\text{Strahler}}\text{'}$: Number of rivers with catchment area ≥ 100 km^2^ estimated by Strahler stream-ordering scheme after correction of the errors induced by pseudo-rivers.

*N*_30_: Number of rivers extracted from the 30 m × 30 m DEMs.

*N*_30(50-100)_: Numbers of rivers with catchment areas 50–100 km^2^.

*N*_30(100-1000)_ are numbers of rivers with catchment areas 100–1000 km^2^.

*N*_90_: Number of rivers extracted from the 90 m × 90 m DEMs.

*N*_900_: Number of rivers extracted from the 900 m × 900 m DEMs.

*N*_900(≥100000)_: Numbers of rivers with catchment areas ≥ 100,000 km^2^ extracted from the 900 m × 900 m DEMs.

*N*_900(10000-100000)_: Numbers of rivers with catchment areas 10,000-100,000 km^2^ extracted from the 900 m × 900 m DEMs.

*N*_900(1000-10000)_: Numbers of rivers with catchment areas 1,000-10,000 km^2^ extracted from the 900 m × 900 m DEMs.

*N*_900(100-1000)_: Numbers of rivers with catchment areas 100-1000 km^2^ extracted from the 900 m × 900 m DEMs.

*N*_900(50-100)_: Numbers of rivers with catchment areas 50-100 km^2^ extracted from the 900 m × 900 m DEMs.

*N*_900(10-50)_: Numbers of rivers with catchment areas 10-50 km^2^ extracted from the 900 m × 900 m DEMs.

*N*_900(10-100)_: Numbers of rivers with catchment areas 10-100 km^2^ extracted from the 900 m × 900 m DEMs.

$\text{N}_{\text{T}}$: Number of rivers with catchment area ≥ 100 km^2^ extrapolated from the number of rivers ≥ 10,000 km^2^.

$\text{N}_{\text{Tmin}}$, $\text{N}_{\text{Tmax}}$: Minimum and maximum values of $\text{N}_{\text{T}}$.

*N*_900_': Number of rivers with catchment area ≥ 100 km^2^ extracted from the 900 m × 900 m DEMs'.

*P*: Mean annual precipitation, mm.

*PET^0^*: Mean annual potential evapo-transpiration, mm.

$\text{P}_{\text{f}}$: Proportions of three stream-ordering schemes (*P*_HO_ : *P*_ST_ : *P*_SH_).

*P*_HO_: Probability of occurrence of segments consistent with the Horton scheme.

$\text{P}_{\text{i}}$: Probability density function, $\text{P}_{\text{i}}\text{=}\int_{\text{Xj-1}}^{\text{Xj}} \text{f}\left( \text{x} \right)\text{dx}$.

$P_{in_{i}}$: Unbiased estimator of *P*_i_ when *n*_i_ is sufficiently large.

$\text{P}_{\text{M}}$: Percentage of migrated rivers.

*P*_SH_: Probability of occurrence of segments consistent with the Shreve scheme.

*P*_ST_: Probability of occurrence of segments consistent with the Strahler scheme.

$\text{R}_{\text{B}}$: Number of rivers at a given order divided by the number at next higher order.

$\text{R}_{\text{B}}\text{'}$, $\text{R}_{\text{B}}\text{''}$: pseudo-bifurcation ratio. The ratio of numbers of rivers with different catchment area grades.

*R*_P_: Ratio of pseudo-rivers.

*S*: Slope (°).

*WO*: Water occurrence.

*X*_1_, *X*_2_, and *X*_3_: Intervals of *f(x).*

*ξ*: Uniformly distributed random variable.

*η*: Random variable, *η* = *f(ξ).*
